# Internally- and externally-driven network transitions as a basis for automatic and strategic processes in semantic priming: theory and experimental validation

**DOI:** 10.3389/fpsyg.2014.00314

**Published:** 2014-04-16

**Authors:** Itamar Lerner, Oren Shriki

**Affiliations:** ^1^Center for Molecular and Behavioral Neuroscience, Rutgers UniversityNewark, NJ, USA; ^2^Department of Brain and Cognitive Sciences, Ben-Gurion University of the NegevBeer-Sheva, Israel

**Keywords:** semantic memory, semantic priming, controlled processes, latching dynamics, neural networks

## Abstract

For the last four decades, semantic priming—the facilitation in recognition of a target word when it follows the presentation of a semantically related prime word—has been a central topic in research of human cognitive processing. Studies have drawn a complex picture of findings which demonstrated the sensitivity of this priming effect to a unique combination of variables, including, but not limited to, the type of relatedness between primes and targets, the prime-target Stimulus Onset Asynchrony (SOA), the relatedness proportion (RP) in the stimuli list and the specific task subjects are required to perform. Automatic processes depending on the activation patterns of semantic representations in memory and controlled strategies adapted by individuals when attempting to maximize their recognition performance have both been implicated in contributing to the results. Lately, we have published a new model of semantic priming that addresses the majority of these findings within one conceptual framework. In our model, semantic memory is depicted as an attractor neural network in which stochastic transitions from one stored pattern to another are continually taking place due to synaptic depression mechanisms. We have shown how such transitions, in combination with a reinforcement-learning rule that adjusts their pace, resemble the classic automatic and controlled processes involved in semantic priming and account for a great number of the findings in the literature. Here, we review the core findings of our model and present new simulations that show how similar principles of parameter-adjustments could account for additional data not addressed in our previous studies, such as the relation between expectancy and inhibition in priming, target frequency and target degradation effects. Finally, we describe two human experiments that validate several key predictions of the model.

## Introduction

One of the most widely investigated phenomena in cognitive psychology is semantic priming. In its essence, semantic priming refers to a simple effect, expressed as the facilitation in recognition of a target word when it is presented in the context of a semantically related cue word (the “prime”) compared to a control condition in which the prime is semantically unrelated (Meyer and Schvaneveldt, 1971). However, more than 40 years of research have shown that this simple effect can be manipulated in various ways to tap a large—and sometimes independent—number of cognitive processes, ranging from semantic activation in memory to encoding of representations, automaticity, word recognition and decision-making (for a review, see Neely, 1991; McNamara, 2005).

Due to the wide range of cognitive processes involved, it is hardly surprising that very different models have been suggested over the years to account for semantic priming, models which have typically referred to only subsets of the whole spectrum of findings in the literature and quite often could not be reconciled with one another. Worse yet, the multiplicity of variables that control the priming effect also tend to interact with each other, leading to a situation in which partial models, successful in accounting for the findings under certain conditions, fail completely under different ones. Consider, for example, the finding that the magnitude of the semantic priming effect is often influenced by the interval between the prime and the target presentation—the Stimulus Onset Asynchrony (SOA). Certain models ascertain that this effect stems from the temporal propagation of semantic activation in memory. These models predict a quick increase in the priming effect, followed by a decrease (Collins and Loftus, 1975; den Heyer and Briand, 1986). Other models assume that strategic processes take place during a priming experiment, either before or after access to the target representation has completed. These models assume priming should mostly increase at long SOAs (Neely, 1977; Becker, 1980; Neely and Keefe, 1989). And yet other models attribute the SOA effect to neuronal properties of the brain structures involved in storing semantic memory, predicting either an increase or decrease, depending on the type of relation between prime and target (Plaut, 1995). As it turns out, all models are right—and wrong—to some degree. The way semantic priming is modulated by SOA depends, in fact, on a specific combination of conditions governing the experiment, from the type of prime-target relation to the type of task used to measure facilitation. Without referring to all of these variables, a coherent picture cannot be drawn. Indeed, one of the most popular models of semantic priming, the 3-process hybrid-model (Neely and Keefe, 1989), quite literally combined together three previous theories, each referring to different aspects of the typical findings, and suggested this hybrid as a “full” account of the phenomenon. Nevertheless, this model, too, had trouble accounting for results indicating the existence of interactions between the various cognitive processes involved (e.g., Balota et al., 1992; Neely et al., 2010).

Recently, we have published a series of computational studies attempting to integrate various results in the semantic priming literature within one, coherent framework (Lerner et al., 2010, 2012a, in press). Basing itself on previous neural-network models of semantic priming, our approach aimed at showing how the addition of several assumptions to the dynamics of a semantic network and the way it is controlled by task-dependent strategies allows fitting a substantial amount of the semantic-priming results in a natural way, and, no less important, demonstrate how various aspects of the relevant cognitive processes interact with each other. Here, we review the main results of these earlier investigations, extend them with two new simulations to entail several findings not addressed before, and present experimental results that are broadly consistent with our approach. Due to limitations of space, we focus on our model; coverage of how it compares to previous accounts of semantic priming can be found elsewhere (e.g., Lerner et al., 2012a).

### Core distinctions relevant to the scope of the model

The literature on semantic priming is vast. Although our model attempts to capture a substantial part of the findings, some aspects are left outside. To begin our review, we first highlight several important distinctions in the priming literature that are relevant to the scope of the model. We focus on distinctions relevant to the most standard version of the task: in each trial, a prime word is visually presented, and, after a certain delay, is followed by a target word. Subjects are instructed to read the prime silently and respond to the target (saying it out loud or deciding if it is a real word or not by pressing one of two buttons) as quickly and accurately as possible while their response-time is measured.

The first distinction relevant to the semantic priming task is the one between automatic and controlled processes. Semantic priming is known to be affected by both. On the one hand, targets are facilitated (i.e., responded to quicker) by their related primes even under conditions that minimize the ability of subjects to carry out complex processing, including when the prime is not consciously conceived (Holander, 1986; Greenwald et al., 1996). Such facilitation is typically taken to reflect interactions between concepts stored in semantic memory that occur beyond the willful intervention of the subject (and thus automatic). On the other hand, priming can also rise or decline as a function of the general task design—for example, when subjects are led to expect, explicitly or though statistical manipulations, that a certain type of targets are about to appear (e.g., Neely, 1977). Target facilitation that is sensitive to such conditions is thought to reflect controlled strategies, employed by subjects attempting to optimize their performance during the experimental session. Our model addresses both types of processing.

A second distinction is between facilitation and inhibition in priming. The fact that related targets are processed faster than unrelated targets could reflect either an actual facilitation of related targets or inhibition (i.e., slowing of response time) of unrelated targets. This is examined by including primes that are considered “neutral” (e.g., instead of a word, a row of X's, see Neely, 1991). If related targets yield shorter Reaction Times (RTs) than neutral targets, facilitation is said to occur. If unrelated targets yield longer RTs than neutral targets, inhibition is exhibited. These effects are not mutually exclusive and can occur in parallel. In practice, semantic priming is known to be composed of, first and foremost, a facilitatory component, whereas inhibition occurs only under certain conditions, usually as a result of controlled processes (McNamara, 2005). Our model is consequently focused on accounting for facilitation, although certain types of inhibition will also be addressed.

Third, there is the issue of which task is utilized to indicate that the target has been recognized. Two tasks have often been used: pronunciation, in which subjects are required to simply say the target word aloud; and Lexical Decision Task (LDT), which requires subjects to decide whether the target is a real word or not and press one of two buttons accordingly (and thus half of the trials consist of pseudo-words as targets). Our model addresses both tasks.

Fourth, there is the issue of pre-lexical and post-lexical processes. Whereas priming can result from cognitive events occurring before the target is recognized, it can also emerge due to processes occurring after the target is recognized but before a response was given. The later processes are sometimes interpreted in terms of controlled decision-making mechanisms and are said to occur only in LDT, in which a decision is needed (e.g., Ratcliff and McKoon, 1988; Neely and Keefe, 1989). Since our model does not contain a decision-making module, only pre-lexical processes are presently addressed.

Finally, there are the various experimental variables that modulate the priming effect: the SOA, the type of prime-target relations, the properties of the stimuli-list used in the experiment, and so on. These are the bread and butter of our model and constitute a large portion of its focus.

In the following, we first present a general description of the model, and then turn to explain how it accounts for various semantic priming effects (more details can be found in Lerner et al., 2012a, in press).

## A latching-dynamics network model of semantic priming

Our account of semantic priming is based on former models that depict semantic memory as a recurrent neural network in which concepts are stored as distributed patterns and form attractors in the network dynamics (e.g., Moss et al., 1994; Masson, 1995; Plaut, 1995; Plaut and Booth, 2000). However, in our model, the network is constantly dynamic: it does not necessarily stay converged on a certain concept for long and tends to jump between attractors due to synaptic depression mechanisms. Moreover, the dynamics is regulated by several parameters that are sensitive to information accumulated during the task and can therefore change the macroscopic behavior of the network throughout the experimental session.

**Figure 1:**
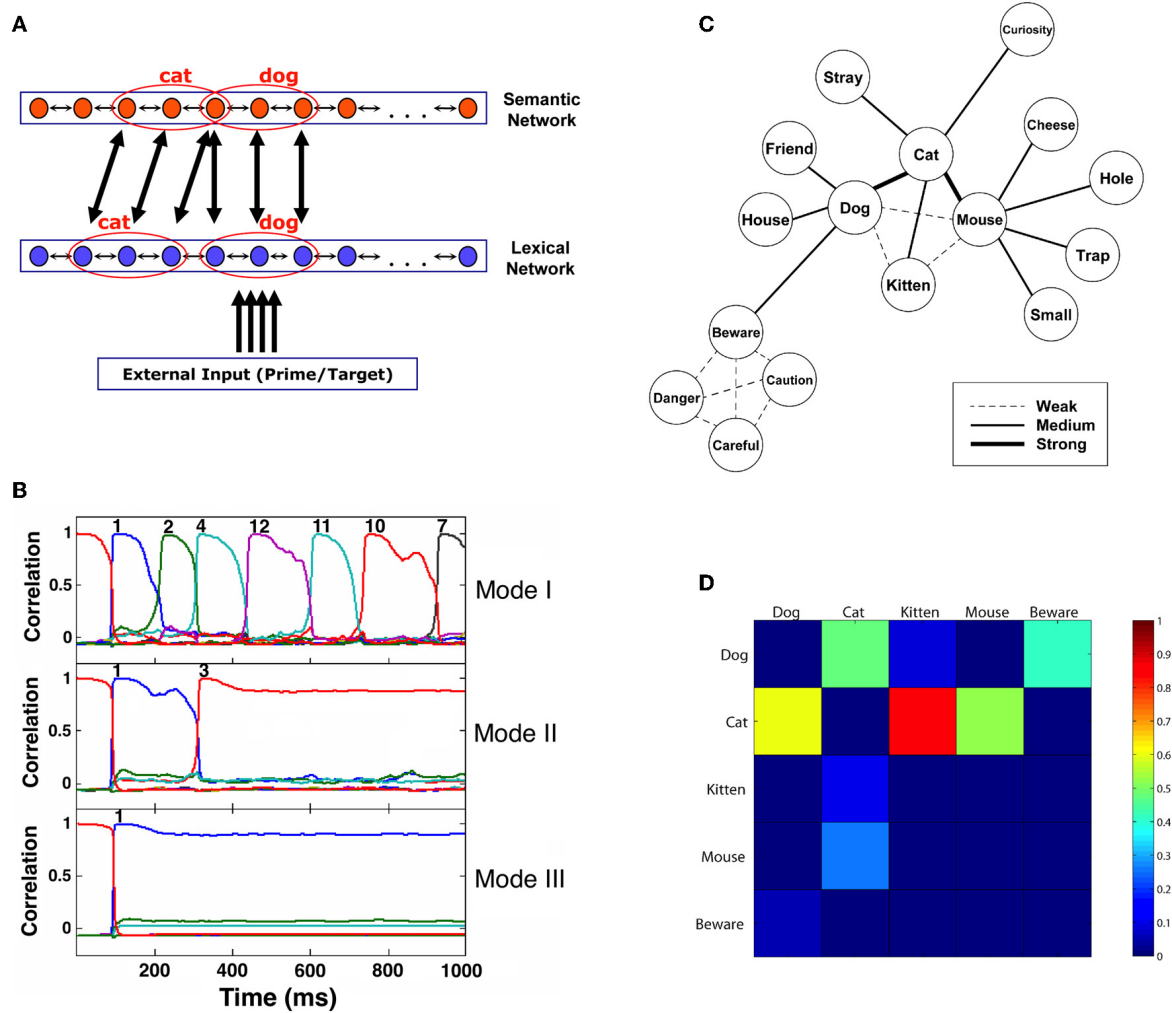
**(A)** Architecture of the network model. Patterns representing related concepts are correlated in the semantic network but uncorrelated in the lexical network. Active units of two toy example patterns representing “dog” and “cat” are marked. Connections between networks are from active units of a pattern in one network to all the corresponding active units in the other network. For simplicity, only some of these connections are drawn. **(B)** Correlation of the semantic network state with its stored memory patterns (representing concepts) as a function of time, showing the differences in typical transitions under various noise values. Each pattern is indicated by a curve with a different color (not all correlation curves are visible at all times, as often they coincide). Convergence to a concept is achieved when a correlation reaches a value of 0.95 or above. The network is presented with an external stimulus corresponding to pattern 1 for 100 ms and then allowed to run freely. Moment of convergence to a specific pattern is indicated by the corresponding pattern number above the appropriate line. Mode I demonstrates dynamics governed by a constant high level of noise. In Mode II, noise is high until the first transition, and then abruptly decreases. Mode III depicts the dynamics when the noise is low throughout the trial. **(C)** Structure of the semantic memory used in some of the simulations. Width of the connecting lines represents the correlation strength between the corresponding concepts. **(D)** First-transition probabilities of the five main concepts in the network from **(C)**. Probabilities are indicated by colors ranging from 0 (dark blue) to 1 (red). Columns represent the presented words and rows represent their associations. (Source: Lerner et al., 2012a; reproduced by permission of Wiley).

In the core of the model are two interconnected computational layers (Figure 1A), representing semantic memory and lexical/phonological memory. Other layers, performing additional processes in word recognition, can be added to this basic structure (see later). Visual input representing a word is assumed to be orthographically analyzed and fed as external input into the lexical/phonologic layer where the word is recognized. The activity elicited in the lexical layer is fed forward to the semantic layer where the word's meaning is stored. Importantly, these processes are bi-directional so that in addition to the feed forward transmission from the lexical to the semantic layer, the semantic layer can influence the lexical layer by feedback (lexical-to-orthographic feedback is addressed at a later stage).

The lexical and semantic layers are modeled as attractor neural networks with sparse representations and continuous-time dynamics (Hopfield, 1982, 1984; Tsodyks, 1990). See Section Materials and Methods for the Neural Network Simulations for full details of the equations and parameters governing the network dynamics. Each network is a fully connected recurrent network composed of 500 units. While the units themselves are analog in the range [0, 1], memory patterns encoded to each network are sparse binary vectors consisting of either fully active (“1”) or inactive (“0”) units. When an external input is fed into units that are part of a specific memory pattern, the activity of the entire network is driven by the internal connectivity to gradually converge to this pattern. The connectivity between the units of each layer is set according to the Hopfield weight matrix for sparse representations, which assures the stability of the patterns. The connectivity between layers and from external sources is always excitatory. Gaussian noise with temporal correlations is added to the local input, inserting some degree of stochasticity to the system. This noise is assumed to reflect, at least partially, signals from interfering brain structures (Zador, 1998; Mato, 1999), and can therefore be attenuated if encouraged to by task conditions.

In the semantic layer, memory patterns represent concepts. As in previous models of semantic memory (e.g., Moss et al., 1994; Masson, 1995), relatedness between concepts is implemented as correlations between memory patterns (reflecting the degree of overlap between them). For example, in Figure 1A, the concepts “*dog*” and “*cat*” are sharing one active unit, making them correlated. The more two concepts are related, the stronger their correlation is; unrelated patterns have a correlation near 0.

In addition to the typical stable-state dynamics, the semantic network is also influenced by synaptic depression mechanisms, which prevent units from maintaining a steady firing rate and make it impossible for the network to sustain its stability infinitely. Such mechanism was previously shown to operate in neocortical synapses (Tsodyks and Markram, 1997) and was also used in connectionist models to account for several experimental findings regarding semantic memory (e.g., Huber and O'Reilly, 2003). In our model, the immediate consequence of synaptic depression is that with time, the network autonomously leaves the present attractor and converges to a different one. The process may repeat again and again, with the network “jumping” from one attractor to another, reflecting what might be seen as a free-association process. This behavior, termed by Treves as “Latching Dynamics” (2005), is characterized by two crucial features: first, there is a higher probability of network transitions between correlated patterns rather than between uncorrelated ones, since the former require fewer changes in the overall activity (Herrmann et al., 1993). Second, transitions depend on the degree of noise in the system: if the noise is very low, the destabilization caused by the depression mechanism would be weak and latching dynamics would not occur; if, however, the noise is sufficiently high, latching dynamics would appear as described (see Figure 1B for examples of the dynamics under different noise values).

In the lexical layer, encoded memory patterns represent words. The dynamics are similar to those governing the semantic network, with two important differences: there are no correlations between the word patterns in the lexical network (indicating no lexical relations between the words, such as “bat”-“rat” and “cable”-“table,” mimicking the lack of such relations in typical stimuli of semantic priming experiments) and there are no depression mechanisms which cause latching dynamics (resulting in simple steady-state behavior with no associative transitions). The links between the lexical and semantic networks are based on connections between active units in corresponding patterns (See Figure 1A). An activated unit in a certain word pattern in the lexical network sends excitatory connections to all active units in the corresponding concept-pattern of the semantic network and vice-versa. Therefore, the activation of one word pattern in the lexical network activates to different extents all related concept patterns in the semantic network, and vice-versa. One exception exists: there is a particular pattern encoded in the semantic network and another encoded in the lexical networks that are not connected to each other (nor are they correlated to any other pattern in their corresponding layer). These patterns, which do not reflect any word or concept, are used as the initial attractors that the networks are converged to at the beginning of each trial in a simulated experiment. Their role is especially important in neutral trials, in which no word pattern is presented as prime; as a consequence, both the lexical and the semantic networks remain converged on these two initial patterns without affecting each other until the target appears (for a thorough discussion of neutral trials and the way to implement them in network models, see Lerner et al., 2012a). Finally, the bottom-up input to the lexical network is also excitatory and activates only the units that are included in a corresponding word pattern.

Lexical-to-semantic connections are strong but are also subject to synaptic depression with slow recovery time. This allows the lexical network to have a fast, short-lived influence on the semantic network, allowing it to quickly converge to the appropriate concept pattern and engage in latching dynamics with no further interference (until a new bottom-up external input arrives and the lexical network converges to a new word pattern). Semantic-to-lexical connections are weak and are not suppressed, allowing the semantic network to have a slow and enduring effect on the lexical network. The asymmetry in the strength of top-down and bottom-up connectivity represents the idea that during semantic priming experiments, subjects' task is to recognize words (a bottom-up process) rather than produce them (see also Stolz and Neely, 1995; Robidoux et al., 2010). This tendency, however, may be reversed if encouraged by task conditions, as discussed later.

### Control over the automatic dynamics

An important assumption in our model is that some global parameters of the network are subject to modulation based on task demands. These modulations represent the neural correlates of controlled processing in semantic priming. One such core parameter is the level of noise in the semantic network, which, as explained earlier, controls the rate of transitions. As discussed by Lerner et al. (in press), the level of noise can be thought of as reflecting the degree of attention given by subjects to the concept that is currently active in memory. Low noise terminates semantic transitions, causing the network to remain converged on (and therefore attending to) the current concept pattern, whereas higher noise levels allow and even accelerate the transitions rate, paralleling a situation in which the subject's thought “flies” from one concept to another without attending long to any particular one. By controlling when transitions are occurring and when they are prevented, the system can decide whether a certain concept is “expected” (i.e., is activated and maintained before the target word appears) or not.

Optimal control of noise is achieved in our network by simple reinforcement learning (see methods in Section Materials and Methods for the Neural Network Simulations; more details can be found in Lerner et al., in press). At the start of an experiment, the noise in the system randomly varies from trial to trial, indicating a “searching mode” of how much attention needs to be given to a stimulus. At some trials, the noise is sufficiently high to allow semantic transitions; at other trials, it is low and does not allow them. The level of noise is also assumed to be modifiable in the middle of a trial, after a transition occurs; in other words, after subjects' thought “wanders,” attention can be re-gathered by reducing noise. Irrespective of the choice of noise, each trial yields an RT to the target, taken to be the convergence time of the lexical network to the corresponding word pattern (see Section How the Model Accounts for Various Findings in Semantic Priming). At the end of each trial, the system compares the current RT to the average RT over all trials experienced so far in the experiment (it is assumed that an approximation of the average RT is accessible to the system, perhaps by using a neural integrator that adds up RTs from the first trial and on as a raw self-evaluation of performance). If the current RT is better (quicker) than the average RT, the particular noise level that was used in this last trial is favored in the next trial. If the current RT was worse (slower) than the average RT, the noise level in next trial tends to change. This way, the system adjusts its level of noise to the level yielding best RTs on average over trials. The result of this process is dependent on the particular type of prime-target relations that are abundant in the stimuli list. For example, sometimes the network is better off avoiding transitions altogether (implemented by keeping a low noise value from the beginning of the trial. This is indicated as “mode III” in Figure 1B). At other times, it is more beneficial for the network to allow a single transition and only then reduce the noise level to cease further transitions (“mode II” in Figure 1B).

Another global network parameter that can be controlled is the magnitude of feedback between the semantic and lexical network (see Brown et al., 2006, for a similar assumption). As previously explained, by default, this feedback is low, emphasizing the bottom-up information processing required in the task. Certain conditions, however, can reverse this tendency and cause the feedback to be more influential. This feedback may be optimized by the same reinforcement mechanism as the noise level.

A final method of control that subjects can establish over the system is expressed as the acquisition of specific episodic associations between concepts, leading to a change in the transition probabilities involving these concepts. For example, subjects can learn that the target word “hand” will likely follow the prime “bird,” adjusting their network transitions accordingly. Such learning, however, does not correspond to the adjustment of a single global parameter.

## How the model accounts for various findings in semantic priming

Consider the basic semantic priming task: a prime word is visually presented, followed (after a certain delay) by a target word. The subject then recognizes the target and his/her response time is measured. In our model, this scenario is reflected by presenting the lexical network with an external input corresponding to the lexical representation of the prime word, and then, following a delay, presenting it with another external input corresponding to the target word. The time it takes the lexical network to converge to the representation of the target word is taken as the response time (neglecting, for simplicity, any decision making processes and motor actions occurring after lexical access). Why does semantic priming emerge in this simple scenario?

When the prime word is presented, the lexical network converges to its corresponding representation and, consequently, sends feed-forward input to the semantic network signaling it to converge to the corresponding semantic representation. The semantic network is then sending feedback to the lexical network. This feedback is weak and does not influence the lexical network immediately. However, when the target word arrives and the lexical network responds by leaving the prime-word attractor and starting to converge to the new target-word representation, the semantic feedback adds up to the bottom-up input and becomes influential. If the prime word was semantically related (i.e., had a correlated semantic representation) to the target word, there is partial congruency between the two (bottom-up and top-down) inputs and this accelerates convergence (see the shared unit in the *dog*/*cat* representations in Figure 1A and its feedback connections to the lexical layer). If, however, the prime and target are not related, the bottom-up and feedback inputs are incongruent and no facilitation occurs. The accelerated convergence in related trials compared to unrelated trials constitutes the semantic priming effect in our model (see Stolz and Besner, 1996, for a similar mechanism in an interactive-activation model). This effect is purely facilitatory: if neutral trials are used (trials in which no word patterns are presented as primes to the lexical network), the resulting RTs are similar to unrelated trials, confirming the lack of inhibition (in fact, had the feedback been strong, inhibition could emerge in the incongruent case. This scenario is discussed later. Nevertheless, under the default assumption of a weak feedback, no inhibition occurs).

While the basic priming effect is easily achieved in the model, the more subtle effects characterizing semantic priming depend on the full dynamics of the model. Specifically, the degree of facilitation that the lexical network will experience depends on the specific concept activated in the semantic network at the time of target onset; and since the semantic network is not static, the transitions it goes through will directly affect this process. The various effects resulting from these dynamics are described below.

### Degree of semantic relatedness and the associative boost effect

One finding in the semantic priming literature is that the stronger the semantic relatedness between two concepts, the larger the priming effect they will produce (Lucas, 2000). In addition, it is known that targets that are not only semantically related to their primes but also associatively related to them (i.e., are often raised as a response to the prime word in a free association test) produce larger priming effects compared to targets that are only semantically related (the so called “associative boost” effect; Moss et al., 1994). Both of these effects are easily achieved in our model.

Stronger priming due to stronger semantic relatedness is readily explained by the fact that semantic relatedness is reflected in our model (as in many former models) as correlations between concept patterns: the stronger the relatedness—the stronger are the correlations. Stronger correlations, in turn, indicate that more shared units are participating in the feedback from the semantic to lexical network, leading to increased facilitation during lexical convergence to the target pattern, hence to more priming.

The associative boost effect is accounted for in our model due to the latching dynamics. Associations between concepts are naturally defined in our network as transitions between concept patterns in the semantic network. A high probability of transition from pattern A to B (e.g., from a pattern representing the concept *dog* to a pattern representing *cat*) thus reflects a strong associative value between the corresponding concepts, mirroring its definition based on free associations. From this definition, two mechanisms give rise to the associative boost effect. The first is straightforward: often, concept patterns that are strongly correlated also yield high probability of transitions between them^1^. Therefore, on average, concepts that are both semantically and associatively related are those that have larger correlations, leading to a larger priming effect. The second mechanism is more directly dependent on the actual transitions: when strongly associated primes and targets are presented to the network in a priming experiment, there is a high probability that the semantic network would jump from the prime's representation to the target's representation even before the visual target actually appears (this can be conceptualized as seeing *dog* and “thinking” about *cat* before the word *cat* is presented). Consequently, when the target is presented, convergence of the lexical network to the corresponding attractor will be strongly facilitated by the feedback from the semantic network due to it being already converged to the target concept. This facilitation is much stronger compared to the case where the semantic network remains converged on the prime or jumps to a concept other than the target, since in the former case all active units will participate in sending feedback; if no such transition occurs, only the units shared by the prime and target will participate in the facilitation. The result is a stronger average priming effect when primes and targets are both semantically and associatively related compared to pairs that are only semantically related^2^.

**Figure 2:**
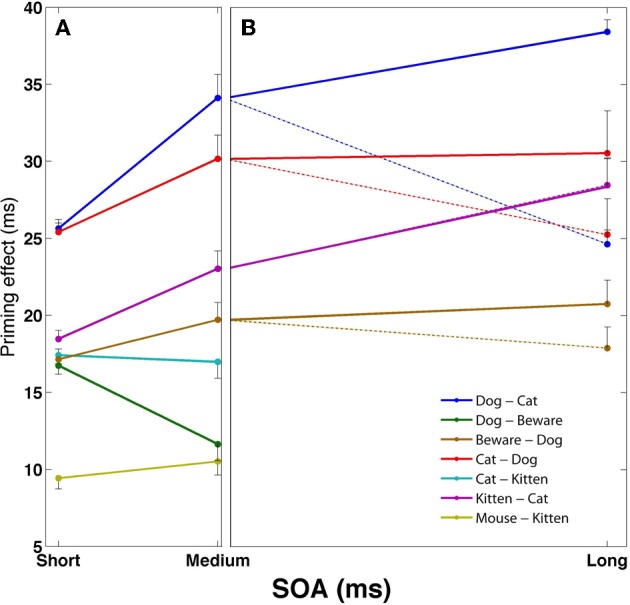
**Mean facilitation effects as a function of SOA for various prime-target pairs using the semantic structure in Figure 1C**. Error bars represent ±1 standard error of the mean. **(A)** SOA effects at short and medium SOAs, under default automatic conditions. **(B)** Long SOA priming effects for the associative pairs from **(A)**, separated to automatic conditions (dotted lines) and controlled-processes conditions in which reinforcement learning of the noise was applied (solid lines).

Figures 1C,D, 2A present the results of a simulation aimed at inquiring how the relations between semantic relatedness, associative strength and SOA are expressed in our model. Sixteen patterns were encoded in the network, each portraying different semantic and associative properties. Figure 1C presents their semantic structure (labeled as words for easier conceptualization), with the strength of the relatedness (i.e., the correlation) between two concepts depicted by the thickness of the line connecting them. Figure 1D describes the associative strength (i.e., the probability of transition) between five of these patterns, based on a free-run of the simulation over 100 repetitions. Figure 2A presents the priming effects for various choices of these patterns as prime-target pairs in a priming simulation. Averaged over SOAs, pairs which are strongly correlated yield stronger priming than pairs which are less correlated (e.g., compare *dog*-*cat, kitten*-*cat*, and *mouse-kitten*); and pairs which are both semantically and associatively related (e.g., *kitten*-*cat*) yield stronger priming than pairs which are semantically related to the same degree but are not associatively related (e.g., *cat*-*kitten*).

### SOA effects

Another classic result in the literature is that semantic priming effects for associated targets increase as the SOA between primes and targets increases, whereas the priming effect of unassociated targets remains roughly the same or even decreases (Plaut, 1995; Lucas, 2000). This finding is replicated in our network due to the latching dynamics. As noted in the previous section, associative prime-target pairs are those for which the network is likely to jump from the prime representation to the target representation. These transitions occur due to synaptic depression mechanisms and their exact time of occurrence is stochastic; however, the probability of a transition increases with time as the depression mechanisms become more and more influential and destabilize the current attractor. In other words, the more time the network stays converged on the prime's concept pattern, the more likely it is to jump out of it. As a consequence, as prime-target SOA increases, it becomes more and more likely that the network would make the transition from the prime to its associated target pattern. The result is an increase in priming with SOA for associatively related targets. The opposite occurs with primes and targets that are not strongly associated. In this case, by definition, the semantic transition is not likely to be to the upcoming target representation. Instead, it is more likely to be to a concept that is either relatively equally related to the target as the prime, or less (e.g., *dog*→*cat*; *dog*→*animal*, etc., when the target is *friend*). Consequently, the priming effect would stay the same or decrease with SOA. Returning to Figures 1D, 2A, now addressing the SOA effect, it is evident that priming of pairs that are strongly associated (e.g., *dog*–*cat*, *cat*–*dog*, *kitten*–*cat*) tends to increase with SOA, whereas priming for unassociated pairs (e.g., *cat*–*kitten, mouse*–*kitten*, *dog-beware*) does not significantly increase, and sometime decreases.

Although the above mechanism neatly accounts for the basic priming dependency on SOA, the actual experimental results are somewhat more complicated and are qualified by additional constraints. Typical findings examining SOA effects under regular conditions have often contrasted priming using associative pairs and priming using category-exemplar pairs (e.g., *fruit*—*apple*). When associative pairs are used, priming increases with SOAs of up to 1000 ms or more. When category-exemplar pairs are used, priming remains roughly the same across SOAs (Neely, 1991)^3^. These priming effects usually endure for at least several seconds, presumingly expressing subjects' attempts to optimize their performance such that semantic activation of the target does not dissipate with time (McNamara, 2005). If, however, the experimental design is carefully shaped to express pure automatic processes, priming may decrease or and even disappear at long SOAs even for associated pairs, expressing dissipation of activation (Neely et al., 2010).

Our model captures this pattern of results by contrasting automatic and controlled processes. Automatic dynamics does not involve any attempt to modulate the noise in the semantic network. At long SOAs, this often leads to the network performing multiple transitions, taking it away from the target representation and reflecting dissipation of target activation. When the noise is optimally modulated, however, the dynamics change significantly. Recall that when reinforcement learning is applied, the network adjusts itself to find noise values that minimize the average RTs to the experimental stimuli. When the stimuli list contains mostly associative pairs, it turns out that RT minimization occurs when a trial begins with a noise value that is sufficiently high to allow one transition, and then decreases to stop further transitions (mode II in Figure 2). This mechanism causes the network to “stay” on the representation it has jumped to (which often corresponds to the upcoming target) and thus allows the priming effect to remain high even at long SOAs such as 1000 ms. Conversely, when the stimuli list contains mostly category-exemplar pairs, RT minimization is achieved when the noise remains low throughout the trial (mode III in Figure 2). This is the case because most category-exemplar pairs are not strongly associated (e.g., *tiger*, *mouse* and *squirrel* are rarely an associate of *animal*) and thus do not benefit from transitions on average. As a result, the network, facing category-exemplar pairs, learns to avoid transitions altogether and remain converged on the prime, yielding a priming effect that is not affected by SOA.

Figure 2B demonstrates some of these dynamics for the strongly associated prime-target pairs of Figure 2A. When the network is allowed to run freely (dotted lines), there are signs of reduction in the priming effect for some of the associated pairs, reversing the increase from short to medium SOAs (longer SOAs reduce the effect even further; see Lerner et al., 2012a). If, however, the reinforcement-learning algorithm is allowed to adjust the noise in the network (solid lines), associated priming effects continue to increase up to the longest SOA without evidence for dissipation of target activation (see similar results for category-exemplar pairs in Lerner et al., in press).

### The relatedness proportion effect

The Relatedness Proportion (RP) refers to the ratio between the number of semantically related trials and unrelated trials within the stimuli list of a semantic priming experiment. A common finding is that a high RP increases priming compared to low RP, but only when the SOA is long (Neely, 1991). Our model accounts for this effect based on how noise modulations are learned to achieve optimal transitions rate.

As was shown in the previous section, the prime-target relationship often determines a “best transitions-strategy” from RT-minimization perspective. For example, when related primes and targets are strongly associated, it is generally favorable for the system, at each trial, to make a transition and remain converged on the concept it has reached. Remaining converged on the prime, in contrast, would not be optimal. Moreover, making more than one transition is also not optimal because it will lead the semantic network to “overshoot” rather than being converged on the most probable target concept (e.g., in a trial where the prime is *dog* and the target is *cat*, it would not be beneficial for the network if two transitions occur during the SOA, such as from *dog* to *cat* and then from *cat* to *milk*). Conversely, if primes and targets are not associated, RT minimization is often achieved by avoiding transitions altogether. The reinforcement-learning algorithm would recognize such tendencies, and, after sufficient amount of trials, push the network to the optimal behavior. However, in order for this learning to occur, the network needs to encounter sufficient amount of related trials consisting of pairs with the relevant relationship. Unrelated trials, in which primes do not hold any predictive information regarding the target, are of no use for the learning mechanism. Therefore, a high RP is required for efficient learning—leading to an increase in the priming effect—whereas a low RP would typically supply the system with insufficient opportunities to extract the regularity. By the same token, short SOAs do not allow sufficient time for transitions to occur frequently, even with high values of noise. Therefore, the system would not be able to learn whether transitions are beneficial simply because it would not experience enough transitions. The result is that at short SOAs, the system would produce the same (suboptimal) RTs whether the RP is high or not. Only when both the RP is sufficiently high and the SOAs are sufficiently long is the optimal transition strategy learned well and priming increases, equivalent to the typical experimental findings.

### Mediated and backward priming

Another well-known finding in the priming literature addresses two specific types of prime-target relations. One, mediated (or “indirect”) relatedness, refers to the case where primes and targets are related only indirectly through a mediating word (e.g., prime: *lion*; target: *stripes*, mediated by *tiger*). The other, backward relatedness, refers to the case where the pairs are associatively related in the opposite direction of presentation, that is, from target to prime but not from prime to target (e.g., prime: *baby*; target: *stork*). Basic findings using these types of pairs show that: (a) Mediated priming can be achieved in pronunciation tasks at various SOAs (and might even slightly rise with SOA), and is always smaller than the direct priming effect (e.g., Balota and Lorch, 1986). (b) Mediated priming does not appear in LDT under typical conditions in which the stimuli list contains both mediated and directly related pairs (“mixed list”); it is, however, evident in LDT if the stimuli list does not contain directly related pairs (“unmixed list”; de Groot, 1983). (c) Backward priming is evident in both LDT and pronunciation using short SOAs and is approximately equivalent in magnitude to forward priming using the same pairs in reverse order (that is, an order which parallels the association, for example: prime: *stork*; target: *baby*; Thompson-Schill et al., 1998). (d) Using longer SOAs, backward priming tends to decrease or even disappear altogether in pronunciation tasks (Peterson and Simpson, 1989; Kahan et al., 1999)—in sharp contrast to forward priming, which increases (e.g., de Groot, 1985). In LDT, however, backward priming is insensitive to SOA (Kahan et al., 1999).

Our model accounts for these results as follows: mediated priming occurs in our network due to transitions from the prime word to the mediating word (e.g., from *lion* to *tiger*). Although the prime in this case is not correlated to the target (corresponding to the lack of direct relations between the concepts), it is strongly correlated to the mediating word, which, in turn, is correlated to the target. When a transition occurs, it is often to the mediating word. Consequently, when the target is presented, the semantic network is converged on a concept that is correlated to that target although the original prime was not. The result is a priming effect which, nevertheless, is smaller on average than direct semantic priming effects due to the fact that the “correct” transition (that is, from prime to the mediating concept) does not always occur, and when it doesn't—the concept that the semantic network ends up converging to is usually not related to the target at all (e.g., *lion*→*mane*, which is not related to *stripes*).

Backward priming occurs in our network based on the assumption that backward related primes and targets have representations which are correlated to each other just like semantically related pairs (a hypothesis shared with former network models of semantic priming, e.g., Plaut and Booth, 2000). Therefore, backward priming occurs at short SOAs from exactly the same reasons as simple semantic priming. Moreover, since correlations are symmetric, it doesn't matter which of the two words is used as prime and which is used as target: both would yield the same priming effect. However, this scenario is true only for short SOAs, where the likelihood of a semantic transition is low. When SOAs are sufficiently long, transitions become probable and therefore the order of presentation becomes significant. By definition, transitions do not occur from prime to target using backward-related pairs. Therefore, as SOA increases, the semantic network would tend to jump from the prime representation to concepts other than the target. Since these concepts would likely be unrelated to the target (e.g., *stork*→*bird*, which is unrelated to *baby*), this mechanism would necessarily reduce or even eliminate the backward priming effect at long SOAs (as an example, compare the backward-related pair *dog-beware* in Figure 2A to the same pair in the forward direction, *beware*-*dog*).

**Figure 3:**
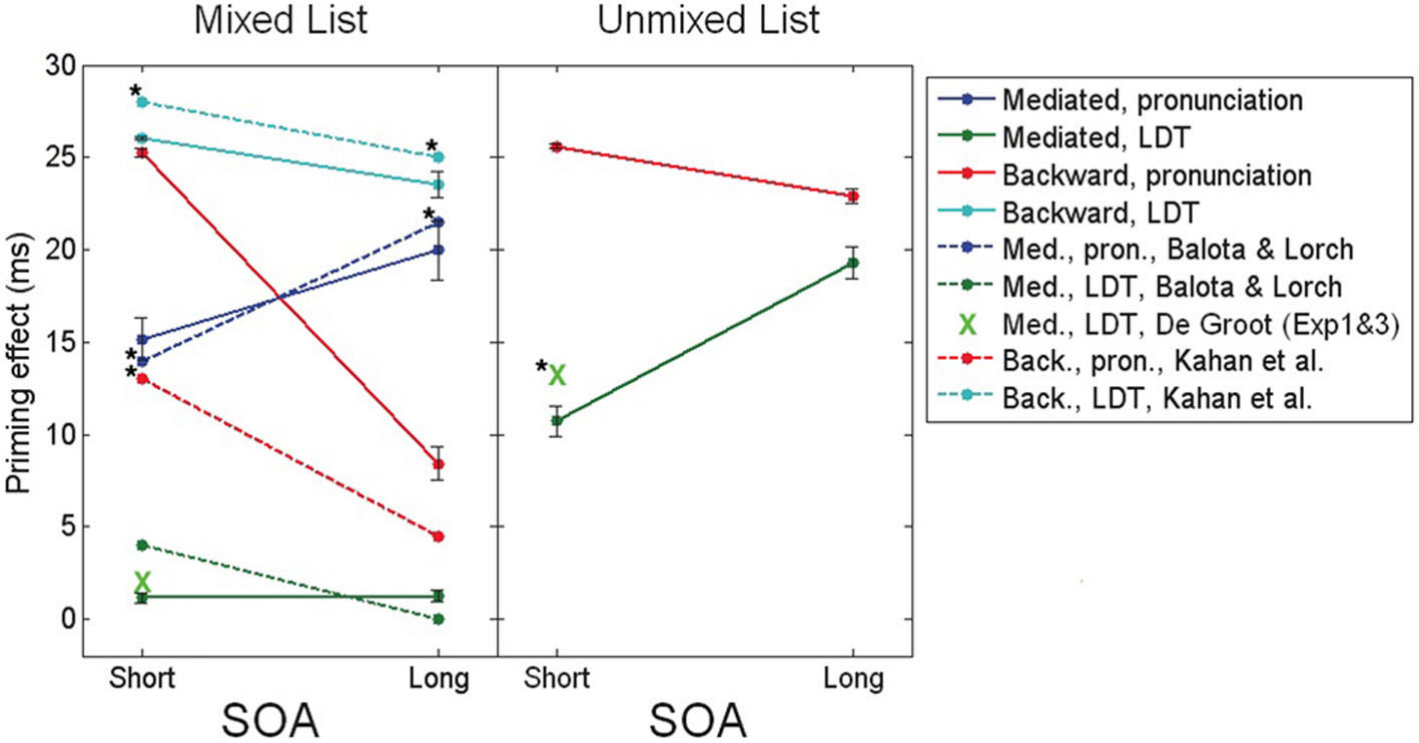
**Mediated and backward priming effects in the model (solid lines), alongside corresponding results from human studies (dashed lines, and green X marks)**. Human results are taken from de Groot (1983), Balota and Lorch (1986), Kahan et al. (1999), with significant priming effects (all at the level of 0.05) marked by a star. Error bars represent ±1 standard error of the mean. (Source: Lerner et al., in press; reproduced by permission of Wiley).

The explanation above portrays a relatively accurate picture of mediated and backward priming in pronunciation tasks. In order to account for the results in LDT, another assumption must be made: whereas in pronunciation the default noise level at the beginning of an experiment is high, allowing semantic transitions to occur, the default noise in LDT is low, preventing transitions. In other words, whereas subjects approach pronunciation tasks in their usual “free association” mode, they approach LDT cautiously, focusing on externally presented stimuli without letting their attention drift (see Lerner et al., in press, for a discussion of this assumption). These default tendencies, however, are simply the initial state of the system and, like always, can be changed with learning. Under this assumption, LDT would not initially permit mediated priming to emerge since this effect depends on transitions. Unmixed stimuli lists, in which all related pairs are in fact mediated-related pairs, push the network to learn that transitions are beneficial: if the network jumps to the right association in a mediated trial, RT would decrease; therefore, the system learns to lift up the noise and allow transitions, leading to the emergence of mediated priming. Mixed lists, in contrast, contain, in addition to mediated pairs, many directly related pairs. Such lists do not necessarily provide a consistent opportunity to learn the benefits of transitions since directly related pairs are not necessarily associated. RTs might increase rather than decrease if the network makes a transition during related trials because the relatedness of the original prime could be lost. Therefore, as a whole, transitions are not necessarily optimal when using mixed lists. Consequently, the system does not learn to increase the noise and no mediated priming emerges. In a sharp contrast, backward priming depends on the lack of transitions. Therefore, this effect typically benefits from the lack of transitions in LDT and would not push the system to change its initial tendency. Backward priming will thus remain stable across SOAs. Figure 3 presents simulation results (taken from Lerner et al., in press, using a different semantic structure than the ones presented earlier) portraying the full spectrum of these priming effects, next to their corresponding results from human studies, showing identical trends. Note also that the explanation above suggests that if a “backward only” unmixed list (containing only backward-related pairs) is to be used in a pronunciation task, the system might learn to avoid transitions (by lowering the noise at the beginning of each trial) and backward priming should emerge even at long SOAs, just like in LDT (Figure 3, right panel, red line). To the best of our knowledge, this prediction has never been tested and remains to be explored in future studies.

## Other controlled effects in the model

The results presented so far have focused on the role of semantic transitions in the network and how they influence automatic and controlled semantic priming. Several known priming results, however, require additional mechanisms that modulate the semantic system: one such mechanism is the influence of subjects' expectancies on the strength of the semantic feedback. Another is the way episodic associations affect transitions in the semantic network. Such mechanisms have already been explored in former models of priming; however, the way they are integrated into our framework provides some new noteworthy findings. These are now presented in some detail.

### Mechanisms

#### Increased semantic feedback

As described above, the feedback from the semantic to the lexical layer in our model was set to be weak compared to the feedforward connectivity. The rationale for this design is that semantic priming experiments do not require, by default, top-down processing. The outcome, however, is that the semantic feedback could not influence the dynamics of the lexical network on its own; only when combined with congruent feed-forward input (as occurs when a related target word is presented as external stimuli) could it make an effect. If, however, these feedback connections were set to be stronger (specifically, above some external threshold characterizing the lexical units), the lexical network could be influenced by the semantic network even before target onset. In a priming experiment, such influence can either accelerate or delay the lexical network's convergence to the target. The acceleration stems from the same mechanisms as in the low-connectivity case: units activated by the feedforward target input are also strengthened by feedback from the semantic layer. A strong feedback may lead this acceleration to be even more pronounced compared to previous cases. The delay, on the other hand, stems from the fact that when feedback is above threshold, some lexical units may receive contradicting inputs: excitation from the semantic feedback and inhibition by the lateral connections coming from other units in the same layer which become active due to the feedforward input. This competition may slow the lexical convergence considerably. When compared to neutral trials (which are not influenced by a stronger feedback since the baseline states of the lexical and semantic network are not connected), significant inhibition may emerge, as opposed to the facilitation-dominant priming of the previous simulations.

**Figure 4:**
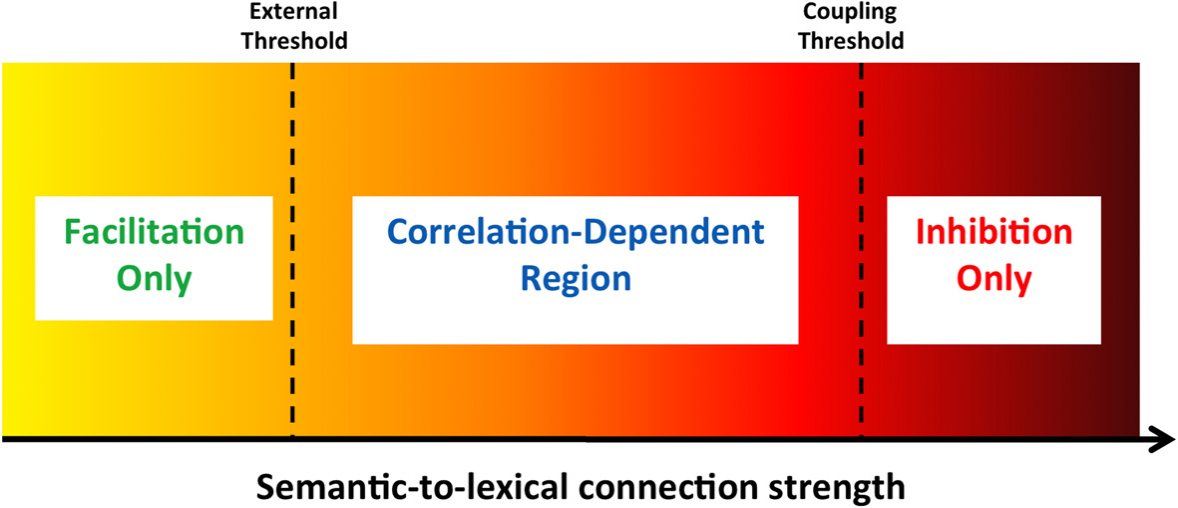
**General regions of facilitation and inhibition as a function of the semantic feedback strength**. Regions are depicted under the assumption that the semantic network is converged to a pattern correlated (but not identical) to the incoming target. With a connection value below the lexical network's external threshold, only facilitation can emerge (as in previous simulations). Connection strength above the “coupling threshold” only allows inhibition. Intermediate connection values cause either facilitation or inhibition, depending on the exact correlation strength. See text for details.

Whether the lexical network's convergence to a target is facilitated or inhibited depends on the specific patterns involved in the process and the strength of the connections. If the semantic network is converged on the concept pattern that exactly corresponds to the upcoming target, lexical convergence to the target will always be facilitated since no contradicting inputs exist. If it is converged on a different and unrelated pattern, lexical convergence will always be inhibited since many units will receive contradicting inputs and no units will receive congruent activation from both the feedforward and feedback inputs. If, however, the semantic network is converged on a pattern that is related—but not identical—to the upcoming target, some lexical units will experience facilitation while others will experience inhibition, with the net influence on convergence time depending on how strong the semantic-to-lexical connectivity is. Figure 4 describes the effective regions of facilitation and inhibition of this latter case as a function of the feedback connectivity strength. Given that the correlations in our network are not very high (due to the coding sparseness), the stronger the connectivity, the more prevalent the inhibition would be. Low connectivity does not reach the lexical units' external threshold and only permits facilitation (as in previous simulations). A level somewhat higher than the threshold still yields facilitation, albeit reduced. With stronger feedback connectivity, inhibition is dominant. The exact connectivity level at which inhibition overcomes facilitation depends on the correlation between the two patterns. Strengthening the connectivity further can cause the lexical network to commit a transition to the corresponding pattern of the semantic network before the external target onset, thus making the lexical and semantic networks coupled. In this case, very strong inhibition will be evident.

From our perspective, one special case of such feedback strengthening is of specific interest: when it occurs as part of an expectancy mechanism. Specifically, previous works have suggested that subjects that expect a certain target to appear may increase the influence of semantic information on lexical processing (Stolz and Neely, 1995; Brown et al., 2006; Robidoux et al., 2010). In our model, this can be manifested as an increase of the semantic feedback gain in parallel to a decrease in the noise level. For example, if a subject expects *cat* to follow *dog*, not only does the noise in the semantic system decrease after a transition to *cat* has occurred (reflecting the expectation for *cat*, as before), but also the strength of the semantic feedback is increased to maximize its influence. This way, if the target is indeed *cat*, all the semantic units active in the representation of the concept *cat* will strongly facilitate the corresponding *cat* units in the lexical layer, causing a great reduction in RT. If, however, the actual target ends up being another word, for example *bark*, the increase in feedback would “make things worse” by inhibiting lexical convergence and elevating RTs. Whether the system will benefit from such feedback increase or is better off without it depends on the specific stimuli list and can be adjusted by reinforcement learning using similar mechanisms to the noise adjustment.

#### Episodic connections

The semantic network's transitions in our model are a product of the correlation structure of the stored concept patterns. This structure is assumed to be rigid and reflects the semantic and associative connections between concepts that were learned during lifetime. However, episodic connections from specific concepts to other concepts can easily be created during day-to-day experiences, as well as in experimental procedures (e.g., Silberman et al., 2005). Such connections do not necessarily reflect the basic semantic relations between concepts and can even be arbitrary. For our model to incorporate transitions between such concepts, the default, correlation-based transition probabilities should be modulated by introducing distinctive connections between memory patterns. Such connections were implemented in previous network models to express episodic relations (cf. Sompolinsky and Kanter, 1986; Herrmann et al., 1993). They require the insertion of additional, unidirectional excitatory synaptic connections between the active units of two concept patterns (see Section Materials and Methods for the Neural Network Simulations). With such connections added, the network, when converged to memory pattern μ, will now have significant probabilities of jumping to memory pattern Υ, even if they are uncorrelated, and these probabilities will increase with the strength of the connections. Other memories will not be affected and the general behavior of the network remains the same. If, in a priming experiment, these two memories are presented as prime and target, a significant priming effect may result. This priming effect will be SOA-dependent because, like mediated priming, it depends on a transition in the semantic network. Many such connections can be added this way and they will all influence the transition probabilities of the network.

### Simulations

#### Facilitation and inhibition in semantic priming and their relation to expectancy

One of the classical findings in the literature regarding expectancy in semantic priming was reported by Neely (1977). In Neely's experiment, subjects' expectations were manipulated such that they expected either prime-related or prime-unrelated targets to appear. When the prime word was the category name “BODY,” subjects were instructed to think about building parts; when it was “BUILDING,” subjects were instructed to think about body parts. When the prime was the category name “BIRD,” subjects were instructed to think about types of birds. Therefore, whereas presenting one of the first two category names created expectations that required a shift from one category to another, presenting the third category name did not require such shift. During the experiment, the expectations were usually fulfilled, which led to facilitation compared to neutral trials, and sometimes unfulfilled, which led to inhibition. However, the pattern of facilitation- inhibition held at long SOAs only. At short SOAs, only facilitation of semantically related targets appeared whereas expectations were inconsequential. These findings were interpreted as supporting the dual effect of a fast semantic activation process based on the structure of semantic memory, accompanied by a slow expectation mechanism.

To simulate the strong expectancies produced in Neely's experiment, we assumed that the network learns both to cease latching dynamics after one transition (as in previous simulations), as well as to strengthen the connections between the semantic and lexical network (indicating a strong reliance on expectancy, hypothesized to occur due to the explicit instructions given to subjects). In addition, the instructional manipulations of the experiment that led subjects to expect unrelated targets were implemented in the network by introducing episodic connections between the relevant unrelated concept patterns. Specifically, the semantic structure of the network consisted of three “neighborhoods,” each containing four moderately correlated concepts (a fourth, unused neighborhood was also encoded, in order to keep the total number of memories similar to previous simulations). These represent the categories BUILDING, BODY, and BIRD, with each concept in a neighborhood representing an exemplar of that category (e.g., *robin*, *sparrow, dove* in the BIRD category). Each concept of the first and second neighborhoods was episodically connected to the concepts of the other neighborhood, thus making transitions between these neighborhoods likely (as in the BUILDING-BODY categories of the human experiment). The third neighborhood, representing the category BIRDS, was left untouched since transitions between patterns within this neighborhood are already very likely due to the correlations between them (adding episodic connections within this neighborhood does not alter our results). In each trial, when a semantic transition occurred, the noise amplitude was reduced to prevent further transitions and the semantic-lexical feedback connections were strengthened (the new connection value was set to approximately the middle of the correlation-dependent region in Figure 4. Since our aim in this simulation was to show how our model implements Neely's results, we did not attempt to fully model a reinforcement-based procedure in which the network learns to reduce the noise and increase feedback, but just assumed that this is the end result of such learning). Trials mirrored Neely's conditions and consisted of either a prime-target pair coming from the third neighborhood (“Non-Shift —Expected – Related” condition), a prime from the third neighborhood and a target from the first two (“Non-Shift—Unexpected—Unrelated” condition), a prime from one of the first two neighborhoods and a target from the other (“Shift—Expected—Unrelated” condition), a prime-target pair coming from within one of the first two neighborhoods (“Shift—Unexpected—Related” condition) or a prime from the first two neighborhoods and a target from the third (“Shift—Unexpected—Unrelated” condition). In addition, neutral trials were carried with a baseline prime and a target randomly coming from any of the three neighborhoods. Each condition was run at 3 SOAs (150, 400, and 700 ms), mirroring Neely's design.^4^

**Figure 5:**
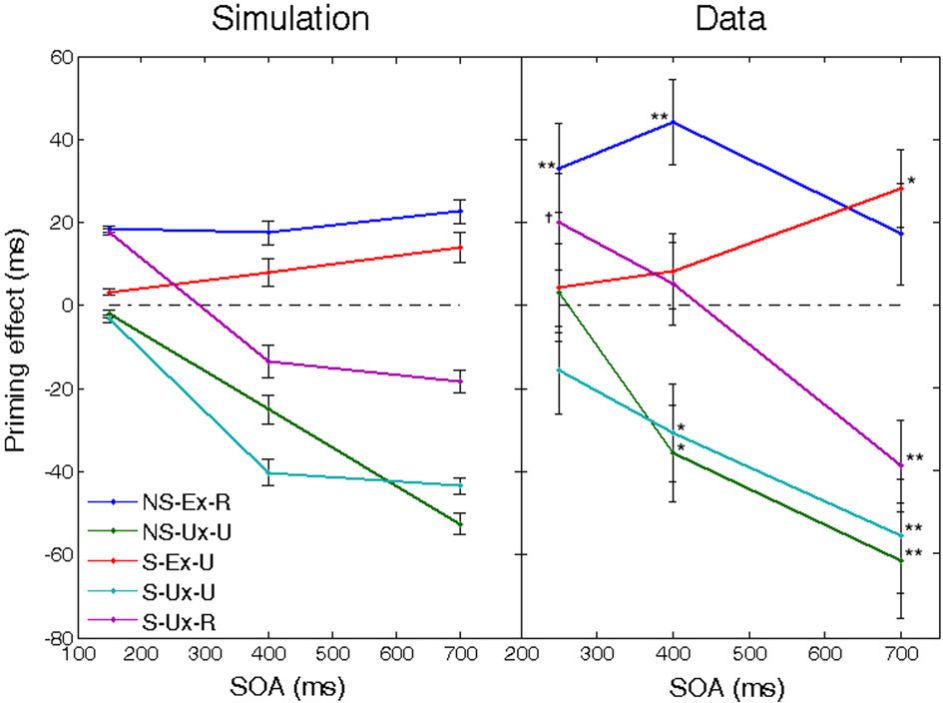
**Priming effects as a function of SOA in a simulation of Neely's (1977) experiment, compared to the original human results**. Positive values indicate facilitation and negative values indicate inhibition, relative to neutral trials. Conditions differentiated on the basis of relatedness (related vs. unrelated targets—R/U), expectancy (expected vs. unexpected targets—Ex/Ux), and whether the expected words required a shift in semantic category (shift vs. non-shift—S/NS). Significant priming effects in the human experiments are marked (†*p* < 0.05; ^*^p < 0.01; ^**^p < 0.001). Error bars represent ±1 standard error of the means. Data of human subjects were taken from Neely (1977). See text for details.

The priming effect (computed relative to the neutral condition) as a function of SOA for each of the five conditions is presented in Figure 5, next to Neely's results. As can be seen, the simulation closely resembled the trends in the human data. Facilitation of related targets was evident at short SOAs, and was maintained at longer SOAs when supported by congruent expectations. When not expected, the recognition of related targets was inhibited at long SOAs. Unrelated but expected targets produced facilitation that significantly increased with SOA. Finally, unrelated and unexpected targets produced an increasing inhibition. All in all, this simulation demonstrates how our model can produce the correct interplay between facilitation and inhibition when modulation of several global network parameters is assumed.

#### Frequency, degradation, and context interactions

Another well-known finding in the priming literature is that the magnitude of priming increases when the linguistic frequency of the target words decreases (Borowsky and Besner, 1993). In addition, priming increases when targets are presented in a degraded form (for example, by lowering the contrast between the letters and the background; Becker and Killion, 1977). These two results are often referred to as the context by frequency and context by stimulus-quality interactions (with “context” referring to the prime-target relations being either related or unrelated). It is also known, however, that frequency and stimulus-quality do not interact (Borowsky and Besner, 1993; Yap and Balota, 2007). Within the interaction-activation framework, which postulates an additional, orthographic layer of computation in which letters are being processed and then sent to the lexical layer, Borowsky and Besner (1993) accounted for these findings in the following way: first, they assumed that high frequency targets have stronger connections from the orthographic to the lexical layer than low-frequency targets (representing the fact that they have been encountered more often during lifetime), and, therefore, their recognition is less influenced by top-down semantic priming. This causes the context by frequency interaction. Second, they assumed that reducing the vividness of the target is equivalent to reducing the external input to the orthographic layer, which in turn delays the bottom-up information from reaching the lexical network. Feedback from the semantic to lexical and from lexical to the orthographic network makes related targets less sensitive to this delay compared to unrelated targets, which induces a context by stimulus-quality interaction. Finally, while context influences both the lexical and the orthographic stages, frequency and stimulus-quality operate at different stages and are therefore additive.

While neatly explaining the results, Borowsky and Besner's account was later challenged by the discovery that the context by stimulus-quality interaction was actually expectancy-dependent (Stolz and Neely, 1995; Brown et al., 2006; Robidoux et al., 2010). However, this dependency could be embedded in their model by assuming that expectancy facilitates the flow of information in the semantic-to-lexical pathway, and without it, this pathway becomes less effective. In order for the context to influence stimulus quality, semantic information must reach the orthographic layer through the lexical layer. Expectancy, by this account, is necessary for transferring the information effectively during the first stage (semantic to lexical) of this route.

**Figure 6:**
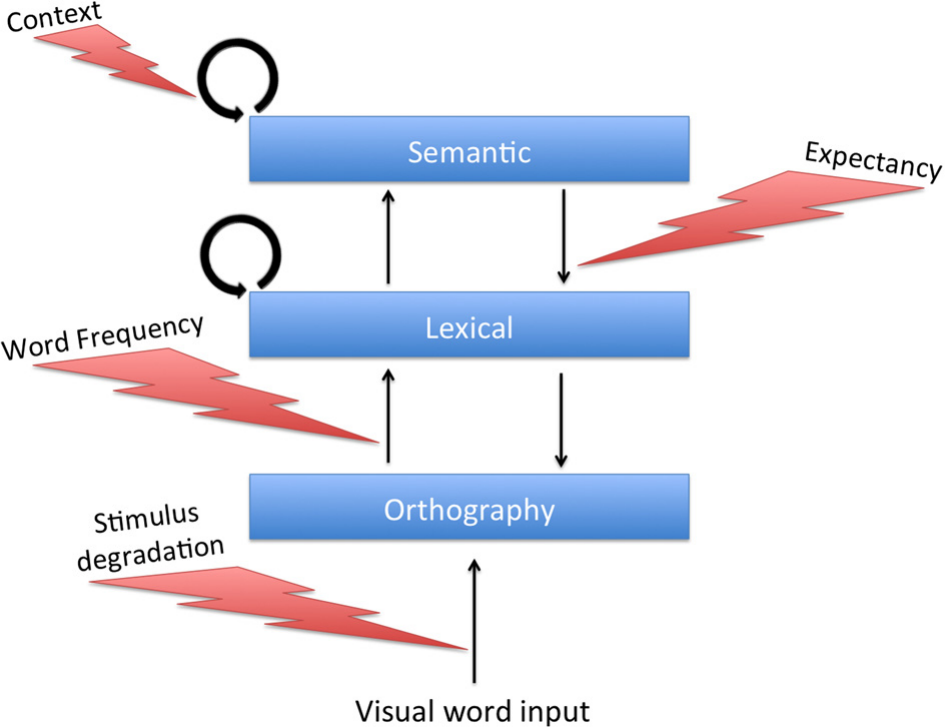
**Model architecture and information processing pathways with the addition of a simple “orthographic” layer**. Task-dependent modulators of specific pathways are marked.

Our model, which bares structural resemblance to interaction-activation models, can account for the influence of frequency and degradation on priming along the lines of Borowsky and Besner's, providing it with a network-based mechanism. Since that model separates between external visual input and orthographic processing, it requires us to add a simple orthographic layer to our model. In our simulations, this layer consisted of 500 additional units that were simple mediators between the external input and the lexical units, and were not laterally connected to each other (see Figure 6).^5^ When one of the patterns was presented to the system, each orthographic unit received the corresponding activity as external input (now representing visual information *per se*) and, after passing some threshold, transferred this information to its corresponding unit in the lexical network through an excitatory connection. These connections were reciprocal and allowed the lexical network to send feedback to the orthographic layer.

The visual input was assumed to rise to its maximal value with some time delay. For clear, non-degraded targets, this delay was extremely short, practically leading to instantaneous rise. For degraded targets, it was significantly longer and postponed the orthographic units' reactions. In addition, the strength of the orthographic-to-lexical connections was set to one of two values, the lower representing low-frequency words and the higher representing high-frequency words (following Borowsky and Besner, 1993). The network was encoded with four semantic neighborhoods, each consisting of four correlated concepts. Prime-target pairs were either related (using any of the correlated pairs, in any order), or they could be unrelated (using concepts from different neighborhoods). Trials were run for each relatedness condition, each frequency value and each stimulus-quality value. Finally, the experiment was run once with the expectancy mechanism active, and once when it was inactive. When the expectancy mechanism was active, noise values were lowered after one semantic transition and the semantic-to-lexical connections were strengthened to a value from the middle of the correlation-dependent region of Figure 4 (as in the previous simulation). When it was inactive, the dynamics were run using the default automatic settings without modulation of noise or feedback.

**Figure 7:**
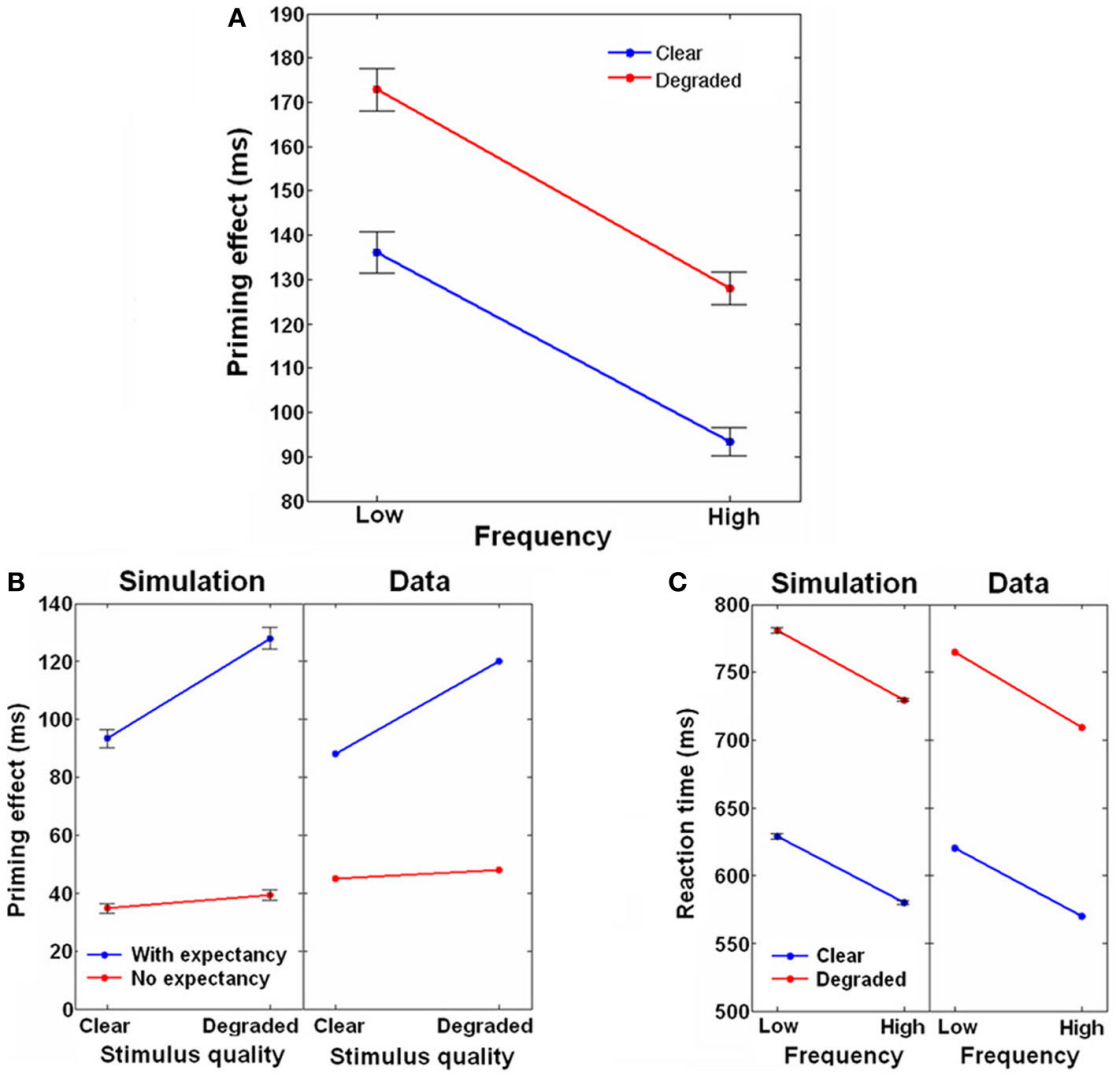
**Joint effects of frequency, stimulus-quality and context. (A)** Priming effect as a function of targets' stimulus-quality (Clear, Degraded) and frequency (Low, High), with expectancy active. **(B)** Effects of stimulus-quality on priming with and without expectancy. Simulation results are compared to human data taken from Stolz and Neely (1995). Data of the simulation are taken from the high frequency condition only. **(C)** Raw reaction times for the frequency and stimulus-quality conditions. Simulation results are compared to human data taken from Yap and Balota (2007). Data of the simulation are taken from the unrelated condition only, when expectancy was active. Error bars represent ±1 standard error of the means.

The priming results for active expectancy are shown in Figure 7A, separated to the different frequency and stimulus-quality conditions. Priming was significantly higher for low frequency targets compared to high frequency targets, and for degraded compared to clear targets. There was no interaction of frequency by stimulus-quality. Figure 7C presents the mean RTs of the unrelated condition compared to the results of an unprimed LDT taken from Yap and Balota (2007) (Since the human RTs include factors like response selection and motor-related delays that are not part of our model, a 450 ms baseline was added to all the simulated RTs to allow comparison). As can be seen, the simulated results closely matched the human findings, with RTs being slower for degraded and low-frequency targets. Again, mirroring the experimental priming results, there was no frequency by stimulus-quality interaction. We further compared how expectancy in our simulation influenced priming of clear and degraded targets of the high frequency condition to the priming of strongly associated pairs taken from Stolz and Neely (1995), who manipulated expectancy through the use of high/low RP. As demonstrated in Figure 7B, similar to the human findings, whereas active expectancy led priming to be affected by stimulus-quality, inactive expectancy eliminated this influence.

Our model is therefore able to replicate the human results concerning the interactions between context, frequency, stimulus-quality and expectancy. The context by frequency interaction emerges, as per the Borowsky and Besner's account, due to the lexical convergence being more affected by semantic feedback when related targets have low frequency (i.e., have lower orthographic-to-lexical connectivity) compared to when they have high frequency. Expectancy is not a prerequisite for this interaction because lexical convergence in the model is affected by semantic feedback even when it is weak, that is, when expectancy is not active. Expectancy, however, is required for the context by stimulus-quality interaction. When expectancy is active, the semantic-to-lexical feedback is increased, feeding the lexical layer with input that passes the lexical units' threshold even before the target appears and thus raising the activity of the corresponding lexical units to a non-zero level. This increase, in turn, raises the local input of the corresponding orthographic units through the lexical-to-orthographic feedback. When a related target appears, this head start accelerates reactions of the orthographic units, countering their delayed reactions to degraded targets. If, on the other hand, an unrelated target appears, the head start cannot assist reactions because different orthographic units are involved; consequently, the degradation-induced delay remains strong. Conversely, when there is no target degradation, both related and unrelated targets are instantaneously recognized by the orthographic layer, making any head start inconsequential. This way, when expectancy is active, context interacts with stimulus-quality (when expectancy is inactive, the feedback from semantic to lexical is below threshold and therefore no lexical to orthographic feedback is transmitted, preventing the context information to affect degradation). Furthermore, once the orthographic units reach their threshold and begin transferring information to the lexical network, stimulus-quality can no longer influence the dynamics, and only frequency (represented by the orthographic-to-lexical connectivity strength) and context (represented by feedback from the semantic network) can have any effect. As a result, stimulus-quality and frequency are additive.

## Experimental support of the model

To conclude our review, we briefly describe two experiments conducted to test some of our model's predictions. The results were largely supportive of the model, although clearly more studies are warranted. Irrespective of our model, our findings pose some serious challenges to former models of semantic priming.

**Figure 8:**
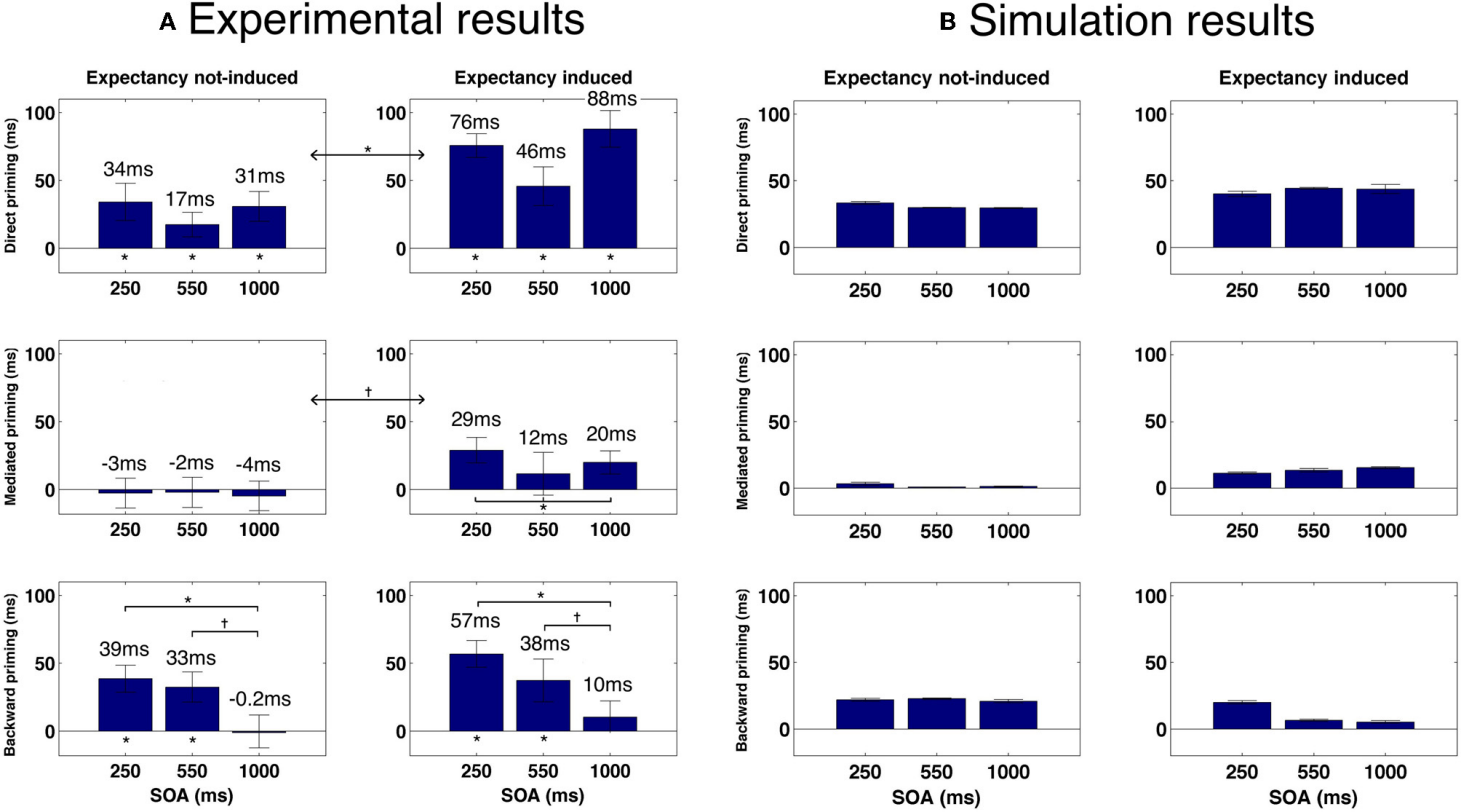
**(A)** Priming results in Experiment 1. Error bars represent ±1 standard error of the mean. † indicates significance level of 0.05 or better; ^*^ indicates significance level of 0.01 or better. **(B)** Simulated priming results corresponding to Experiment 1 under equivalent conditions.

One important prediction that comes out of our model is that mediated priming should occur in LDT if expectancies are manipulated to make semantic transitions highly beneficial. Recall that in LDT, stimuli lists that contain both direct and mediated pairs do not yield mediated priming effects whereas when the stimuli list contains only mediated pairs (unmixed list), mediated priming does appear. According to our model, this is because unmixed lists allow the system to consistently experience trials in which transitions are beneficial (mediated priming can emerge only by transitions, in contrast to direct priming), thus allowing it to overcome the initial tendency to avoid transitions. Continuing with the same rationale, a natural prediction would be that a mixed stimuli list in which related pairs are chosen such that they will also consistently benefit from transitions, should yield mediated priming as well. By the same token, the model predicts that using such mixed lists in LDT should cause a reduction in backward priming with SOA (contrary to typical LDT experiments in which backward priming is relatively insensitive to SOA, e.g., Kahan et al., 1999). The reason is the same: if related pairs strongly encourage transitions, the system would learn to perform transitions in every trial. As a result, backward-related primes, which depend on prime-target correlations, will lose their impact when the SOA is sufficiently long. To sum up: the model predicts emergence of mediated priming, and a decrease in backward priming with SOA, in LDT using a mixed list which consists of many directly-related primes that benefit from transitions (e.g., strongly-associated primes and targets). When the mixed list does not contain many such related pairs, mediated priming should not appear and backward priming should persist across SOA (see Figure 8B for simulated results demonstrating these predictions).

**Figure 9:**
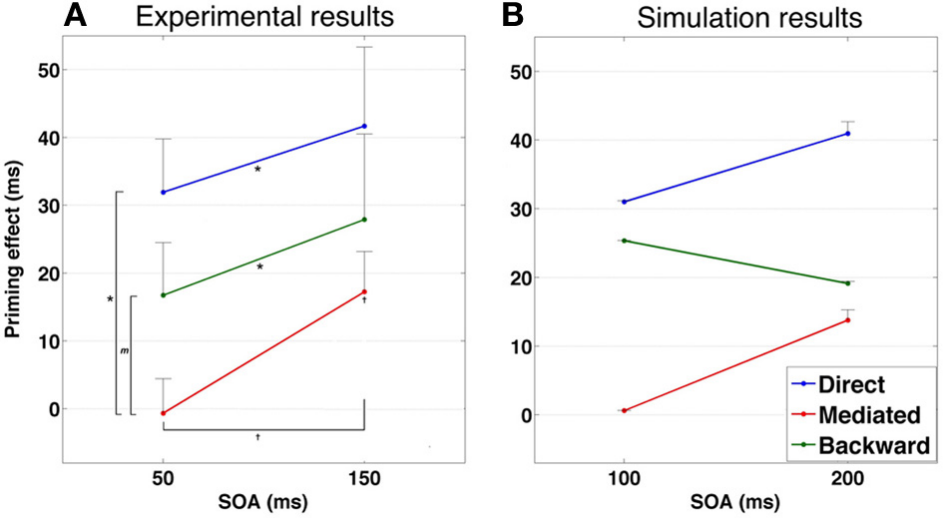
**(A)** Priming results in Experiment 2. Error bars represent ±1 standard error of the mean. † indicates significance level of 0.05 or better; ^*^ indicates significance level of 0.01 or better. *m* indicates a marginally significant result (*p* < 0.08). **(B)** Simulated priming results corresponding to Experiment 2 under similar condition.

A second prediction relates to the existence of mediated-priming at extremely short SOAs. Since mediated priming depends on transitions, and since transitions take time to occur, using very short SOAs should abolish mediated priming even if the stimuli list encourages it. This prediction was, in fact, explored in the past (Ratcliff and McKoon, 1981) and results did not support it. However, there were several confounds in this past study (for a discussion, see Lerner et al., 2012a), making it difficult to draw a definite conclusion. One example of a possible confound is that very short SOAs by themselves might not be sufficient to limit processing time of the prime before target recognition (den Heyer and Briand, 1986). Subjects might simply delay target processing even after it appears on screen, effectively lengthening the SOA. One method commonly used to avoid such confound is using forward masking of the primes (Perea and Rosa, 2002). Such procedure, combined with brief SOAs, typically prevents conscious recognition of the prime and emphasizes automatic processing over strategic ones. Our model therefore predicts that using brief SOAs together with forward masking of the prime should prevent mediated priming. In contrast, backward and direct priming should persist under these conditions since both depend on direct correlations between primes and target and do not require transitions (see Figure 9B for simulated results demonstrating these predictions).

In the following, Experiment 1 tested our first prediction and Experiment 2—the second prediction.

### Experiment 1

#### Methods

***Participants***. One hundred and forty-four undergraduate students at the Hebrew University of Jerusalem participated in the experiment for credit or payment. All were native Hebrew speakers. The 144 participants were randomly assigned to one of the six experimental groups (see design), with 24 participants in each group. There were no conspicuous differences between the groups regarding the participants' handedness, gender, age or education.

***Design***. Direct, mediated and backward-related priming effects were tested in a 3 × 2 between-subjects design. The between-subjects factors were SOA (250, 550, 1000 ms) and Expectancy (induced, not induced). Although the direct, indirect (mediated) and backward-related pairs were mixed within-subject and presented in random order in one block, these effects were not directly compared to one another since we were not interested in making a priori predictions regarding differences across priming conditions, and different targets were used in each condition (see stimuli)^6^. The dependent variable for each analysis was the priming effect, calculated for each subject as: Median(RT)_unrelated_ − Median(RT)_related_, and all subsequent statistical analysis was computed based on this variable.

***Stimuli***. The word-targets used in Experiment 1 (hereafter, the “experimental” targets) were 80 different Hebrew words (with the vast majority being nouns and adjectives in singular form), out of which 26 words belonged to the directly-related condition, 30 to the mediated condition and 24 to the backward-related condition (the number of targets for each condition was determined according to the availability of suitable primes in the Hebrew norms). Each of these targets was paired with a related word according to the relatedness condition it belonged to. Relatedness in each group was based on published Hebrew association norms (Rubinsten et al., 2005), and were determined as follows: the primes in directly related pairs had a strong forward association to their targets (mean association value = 0.38, range: 0.32–0.41. The scale in these norms is 0–1, but association strengths of 0.3 and above are already considered very strong). The primes in indirectly related pairs (the mediated priming condition) were strongly associated with a word that has not been actually presented (the mediator), which, in turn, was strongly associated with the target. Importantly, no direct forward or backward association existed between the prime and the target in such pairs (mean association value = 0). In addition, the absence of direct semantic relatedness between these primes and targets was verified in a pilot experiment with participants who were not tested in the main experiment. In the pilot, participants were instructed to rate the semantic relatedness of the 80 relevant prime-target pairs used in this study on a scale from 1 (not related) to 7 (highly related). The mean rating of the pairs included in the mediated priming condition was a low 2.07 (range: 1.12–3). Finally, backward-related pairs were constructed of words which had no forward association based on the norms but had a medium-to-strong backward association (mean backward association value: 0.33; range: 0.07–0.82), and received a low semantic relatedness rating in the pilot test (mean: 2.76; range 2.17–3.46).

For each of the three related-pairs lists, a control list was constructed in which the pairing between the primes and the targets in the same condition have been pseudo-randomly shuffled such that the new pairs were semantically unrelated. This procedure yielded three unrelated lists corresponding to the three related conditions. During the experiment, half of the experimental targets in each relatedness condition were presented with their related primes and half were presented with their unrelated primes using a counterbalanced design, ensuring that although no word was repeated within-subject, across participants all targets appeared equally in the related and in the unrelated condition.

In addition to the experimental words, 280 filler word-pairs were assembled. Among these pairs, 140 were very strongly associated (based on association norms), 30 were related as category-exemplars, and 110 were unrelated. All of these words were different than the experimental words. The filler pairs were used to manipulate the RP and the prevalent type of prime-target relations in the experimental list, which are assumed to affect expectancy. In the expectancy-induced condition, the 140 strongly associated filler pairs were added to the experimental pairs. In the expectancy not-induced condition, the 30 categorically-related and 110 unrelated pairs were added to the experimental pairs.

Finally, there were also 220 pseudoword-targets, all paired with word-primes. The pseudowords were phonologically legal derivations of real words in which one syllable was changed to render a meaningless item. The primes paired with the pseudowords were unrelated to the original word from which the respective targets have been constructed (and were also different than the experimental and filler words).

***Procedure***. The experiment was run in a quiet testing-room on a PC computer using the E-Prime software (version 1.1). All stimuli appeared on screen in black over a white background in David font type size 40. Participants sat approximately 60 cm from the screen with their right and left index fingers resting on the “L” and “A” keyboard keys, respectively. At the beginning of the session, subjects read written instructions on the screen describing the LDT. The instructions informed the participant that in each trial, the target stimulus is preceded by a briefly presented word that should be read silently but to which no overt response is required. Participants were instructed that the lexical decision to the target should be reported by clicking on the “A” key for a “word” response and on the “L” key for a “nonword” response. Speed and accuracy were equally emphasized.

After making sure the instructions were clear, subjects performed a 22-trials practice session that was identical in structure to the experimental session, but contained different word pairs. If performance during training was not adequate (a rare event), the experimenter could repeat the practice phase until it was clear that the subject had mastered the task. After practice, the experimenter left the room and the experimental phase initiated. The 440 trials were presented in 3 (nearly) equal blocks with two short breaks in between (the duration of which was controlled by the subject).

In each trial, a small cross-shaped fixation cue appeared on screen for 700 ms and then replaced by the prime word, which remained visible for a constant duration of 250 ms. After an Inter Stimulus Interval (ISI) of 0, 300, or 750 ms (depending on the SOA condition) the target appeared and remained on screen until the subject's response. Thus, the SOA was 250, 550, or 1000 ms. During the ISI between the prime and the target, the screen was blank. An inter-trial interval of 2000 ms followed the response. All stimuli occurred randomly throughout the test list.

#### Results

The median RTs in each experimental condition are presented in Table 1. As evident in that table, in most cases the RTs in the related conditions were faster than in the unrelated conditions, demonstrating priming effects of different magnitudes. The most notable exception was in the mediated priming condition when expectancy was not induced.

**Table 1:** **Mean reaction times across subjects (ms) in Experiment 1^7^**.

		**Direct**		**Mediated**		**Backward**	
		**Induced**	**Not induced**	**Induced**	**Not induced**	**Induced**	**Not induced**
250	Related	489	548	531	568	537	544
	Unrelated	565	582	560	565	594	583
	Priming	76	34	29	−3	57	39
550	Related	518	507	551	525	569	517
	Unrelated	564	524	563	523	607	550
	Priming	46	17	12	−2	38	33
1000	Related	467	533	519	564	542	560
	Unrelated	555	564	539	560	552	560
	Priming	88	31	20	−4	10	0

For statistical analysis, the priming effect was calculated for each subject and each priming condition (direct, mediated and backward) as the median RT to related targets subtracted from the median RT to the corresponding unrelated targets. Only correct responses were used, thus excluding 3.18, 3.37, and 5.3% of the responses in the direct, mediated and backward priming conditions, respectively. Each priming-type condition was assessed separately using Analyses-of-Variance (ANVOA) with Expectancy (induced, not induced) and SOA (250, 550, 1000) as between-subjects, independent factors^8^. These priming effects are presented in Figure 8, alongside corresponding simulated results with equivalent conditions (the semantic structure used in this simulation was taken from Lerner et al., in press, similarly to the simulation results of Figure 3). As seen in the figure, the magnitude of priming was differently affected by SOA and expectancy across the various types of relatedness.

In the direct priming condition, ANOVA showed that the priming effect was larger in the expectancy induced than in the non-induced condition [*F*_(1, 138)_ = 18.8, *MSE* = 3417.2, *p* < 0.001]. There was also a significant main effect of SOA [*F*_2, 138)_ = 3.1, *MSE* = 3417.2, *p* < 0.05], but no interaction between these two factors (*F* < 1). Pairwise comparisons (Bonferroni-corrected) showed that the priming effect was smaller in the 550 ms SOA condition than in the 1000 ms SOA condition, an effect that was marginally significant (*p* = 0.064). Priming was not significantly different, however, when comparing the 250 ms SOA condition to either the 500 ms or the 1000 ms SOA conditions. (both *p*s > 0.1). Individual *t*-tests of the priming effect for each SOA separately (collapsed over the two expectancy conditions) showed that for all three SOAs the direct priming effect was significantly higher than zero [*M* = 55 ms, *t*_(47)_ = 6.42, *p* < 0.001; *M* = 31.7 ms, *t*_(47)_ = 3.67, *p* < 0.002; *M* = 59.4 ms, *t*_(47)_ = 6.19, *p* < 0.001, for the 250, 550, and 1000 ms SOAs, respectively; Bonferroni-corrected]. Similar *T*-tests of the priming effect for each expectancy condition separately (collapsed over SOA) showed that under both expectancy conditions priming was significant [*M* = 27.6 ms, *t*_(71)_ = 4.21, for the expectancy not-induced condition; *M* = 69.8 ms, *t*_(71)_ = 9.45 for the expectancy induced conditions. *p* < 0.001 for all comparisons, Bonferroni-corrected].

In the mediated priming condition, only the main effect of expectancy was significant [*F*_1, 138)_ = 6.3, *MSE* = 3090.3, *p* < 0.02; all other *p*s > 0.7] indicating higher priming effect in the expectancy induced than in the not-induced condition. Collapsing over SOA, separate *t*-tests (Bonferroni corrected) showed that mediated priming was significant when expectancy was induced [*M* = 20.3 ms, *t*_(71)_ = 3.03, *p* < 0.007] but not when expectancy was not induced (*M* = −3.1 ms, *p* = 0.62).

In the backward priming condition, only the main effect of SOA was significant [*F*_2, 138)_ = 6.7, *MSE* = 3428.9, *p* < 0.003]. Collapsing over expectancy conditions, Bonferroni-corrected pairwise comparisons showed that backward priming was significantly smaller for the 1000 ms SOA compared to either the 250 ms SOA condition (*p* < 0.002) or the 550 ms condition (*p* < 0.05). There was no significant difference between the 250 and 550 ms SOAs. Separate Bonferroni-corrected *t*-tests for each SOA condition showed that the priming effect was significantly larger than zero for the 250 ms [*M* = 47.8 ms, *t*_(47)_ = 6.78, *p* < 0.001] and 550 ms [*M* = 34.9 ms, *t*_(47)_ = 3.65, *p* < 0.002] conditions, but not for the 1000 ms SOA condition (*M* = 5.1 ms, *p* = 0.55).

No error-analysis was attempted due to the small number of errors (often zero) made by the majority of participants.

### Experiment 2

#### Methods

***Participants***. Fifty-two native-Hebrew speakers, undergraduate students at the Hebrew University of Jerusalem participated in the experiment for credit or payment. They were randomly assigned to two SOA groups with a total of 24 participants in the first group and 28 in the second.

***Stimuli and design***. The same stimuli of the expectancy-induced group from Experiment 1 were used, with Priming-type (Direct, Mediated, Backward) as a within-subjects factor. The only between-subjects factor was SOA (50, 150 ms; cf. Ratcliff and McKoon, 1981).

***Procedure***. The procedure was similar to Experiment 1, with one difference: instead of a small fixation cue in the middle of the screen, the pattern “#######” was used, extended to cover the whole space on which the prime and target words were to appear and effectively inducing forward-masking on the prime. As previously explained, this change was made to minimize awareness to the prime word, thus reducing the possibility that subjects are holding back target-processing until the conclusion of prime processing (a situation that diminishes the effectiveness of the SOA manipulation; see Perea and Rosa, 2002).

#### Results

Median RTs in each experimental condition are presented in Table 2. As in Experiment 1, the median RTs of the related pairs in all but the short-SOA mediated-priming condition were faster compared to the unrelated pairs.

**Table 2:** **Mean reaction times across subjects (ms) in Experiment 2**.

		**Direct**	**Mediated**	**Backward**
50	Related	550	560	574
	Unrelated	582	559	590
	Priming	32	−1	16
150	Related	533	537	561
	Unrelated	575	555	588
	Priming	42	18	27

Erroneous answers (constituting 5.1, 3.72, and 5.69% of the total responses in the direct, mediated and backward conditions, respectively) were removed from further analysis. Repeated measures ANOVA with Priming-type (Direct, Mediated, Backward) as within-subjects factor and SOA (50, 150) as between-subjects factor revealed a main effect of Priming-type [*F*_2, 100)_ = 6.1, *MSE* = 1722.7, *p* < 0.04], and, at a trend level, main effect of SOA [*F*_1, 50)_ = 2.7, *MSE* = 807.9, *p* = 0.108]. The interaction between these factors was not significant (*F* < 1).

Next, we analyzed how priming-type affected priming separately for the two SOAs. Repeated-measures ANOVA showed that priming-type significantly modulated the priming effect [*F*_2, 54)_ = 6.1, *MSE* = 1217.1, *p* < 0.005] at 50 ms SOA, but not at 150 ms SOA (*F* = 1.6, *p* = 0.221). Planned comparisons at the 50 ms SOA condition showed that mediated priming was significantly smaller than direct priming (*p* < 0.003) and was also smaller than backward priming, although the latter effect was only marginally significant (*p* = 0.073).

We then analyzed each priming type separately, as in Experiment 1. ANOVA revealed that SOA did not modulate either direct or backward priming (both *F*s < 1). Therefore, for both priming types, the data was collapsed over SOA. Separate *t*-tests showed that both effects were significantly above zero [*M* = 21.9 ms, *t*_(51)_ = 3.1, *p* < 0.007; *M* = 36.4 ms, *t*_(51)_ = 5.4, *p* < 0.001, for direct and backward priming, respectively; Bonferroni-corrected), indicating the existence of both direct and backward priming at these short SOAs. ANOVA revealed, however, that SOA did modulate the mediated priming effect [*F*_1, 50)_ = 5.3, *MSE* = 777.6, *p* < 0.03]. The significance of this effect was therefore examined separately at each SOA. Individual *t*-tests showed that mediated priming was significant for the 150 ms SOA condition [*M* = 17.2 ms, *t*_(49)_ = 2.9, *p* < 0.02] but not for the 50 ms condition (*M* = −0.7 ms, *p* = 0.9). These results are plotted in Figure 9, alongside corresponding simulated results with equivalent conditions^9^.

### Discussion

The two experiments confirmed most of our model's novel predictions: using a stimuli list with a high proportion of strongly associated pairs, assumingly encouraging transitions, we have found mediated priming at all SOAs; in contrast, a low proportion of category-exemplar pairs (assumingly discouraging transitions) did not result in a significant mediated priming effect in any SOA. Moreover, using a high proportion of associated pairs did not yield mediated priming when the SOA was very brief and forward masking was employed. Also as predicted, when using the associative-pairs list, backward priming decreased as SOA increased from 250 to 1000 ms and, furthermore, was evident even at very brief SOAs, exactly as direct priming. These results support the crucial conjectures made by our model regarding the involvement of semantic transitions in the elicitation and temporal development of the semantic priming effect in general, and of mediated and backward priming effects in particular.

Contrary to our prediction, backward priming decreased with SOA even when using the category-exemplar list. This last result stands in contrast not only to our model, but also to typical findings using backward priming in LDT (Kahan et al., 1999; McNamara, 2005). More experiments should therefore be performed in order to clarify this issue. Similarly, there was also some discrepancy between current and previous human results (and the simulation) concerning direct priming effects: whereas direct priming usually tends to either increase or maintain its magnitude with SOA (Lucas, 2000), our results yielded an unexpected decrease in direct priming at the 550 ms SOA compared to the 250 and 1000 ms SOAs. Given its uncharacteristic nature, it is not clear how to interpret this finding; however, since our main focus in the current experiments was mediated and backward priming rather than direct priming, we do not see this result as posing a crucial challenge to our model. Finally, another small discrepancy appeared in the backward priming results of Experiment 2: whereas there was no statistical difference in backward priming between 50 and 150 ms, the corresponding simulation results showed backward priming has already started decreasing at the longer (200 ms) SOA. However, this may be explained by small differences in the timing of transitions; taking Experiments 1 and 2 together, it is clear that the human results also show a decrease in backward priming with SOA; therefore, the decrease shown in the second simulation is also evident in the human results but at a slightly longer SOA.

Our experimental results are consequential not only in relation to our model, but also to previous models of semantic priming. First, to the best of our knowledge, they constitute the first evidence that mediated priming is influenced by expectancy- modulators such as RP. Traditional views have treated mediated priming as resulting from pure automatic mechanisms and, in fact, assumed that controlled processes abolish this effect (Neely, 1991). Given our results, this is clearly not the case (see Jones, 2010, 2012, for related results). Second, two prominent studies in the past (Ratcliff and McKoon, 1981; Lorch, 1982) have determined that the onset of mediated priming parallels that of direct priming. Their findings have been influential in shaping several models of semantic activation (e.g., ACT-R. Anderson, 1983; Jones et al., 2006). Our results cast doubt on this claim, proposing instead that the former studies did not control for several important factors related to mediated priming (see discussion in Lerner et al., 2012a). We view this issue as central to models of semantic memory, and one that requires more investigation in future studies. In addition, some predictions of our model are yet to be tested: for example, our model predicts that using an unmixed list of backward-priming pairs in a pronunciation task should eliminate the SOA dependency of this effect (see Lerner et al., in press, for details). Future studies may test this prediction as well.

## Concluding remarks

In this review we attempted to give a general and concise description of our recent neural network model of semantic memory, which was designed to account for various findings in the semantic priming literature within a unified framework. We have shown how the model can account for some of the core results in the literature, and also provided some experimental support to several of its main conjectures. Although the model is based on one central claim, namely, that semantic activation is a result of an interplay between correlations of encoded concept representations and transitions between these representations, some of the model's explanatory power derives from additional assumptions: we assumed that the rate and type of transitions can be modulated by attentional (“noise”) shifts, episodic learning and task type, and that the feedback from the semantic to lexical layer is subject to expectancies. These additional assumptions reflect the many levels of processing that contribute to the semantic priming phenomenon. Nevertheless, many of our assumptions have already been raised in previous models based on experimental evidence, and the way they are combined in our model gives them additional explanatory strength.

It is important to acknowledge some of the limitations of our model in its current form (see also Lerner et al., 2012a). One limitation is that the model, which is based on Hopfield-like connectivity, does not constitute a working-memory system; consequently, the network cannot fully activate more than one stored item at each moment in time (although partial activation of several items in parallel does occur due to the correlations between patterns). This limits the model's ability to yield concurrent priming effects of multiple targets (e.g., Balota and Paul, 1996; Lavigne et al., 2011). In addition, although Hopfield connectivity asserts that memory patterns are encoded using a Hebbian learning mechanism, the exact way by which concepts are learned and stored is not addressed, and some of the biological assumptions of Hopfield networks are unrealistic; it is therefore an open question to what degree our ideal semantic neighborhoods can represent the real structure of semantic memory. Nevertheless, the core principles of the model, emphasizing the contribution of synaptic depression to dynamical changes in activation of concepts over time, are independent of the particular network implementation; therefore they can serve as explanatory mechanisms in future network models of semantic priming based on different connectivity principles which are more biologically realistic (e.g., Brunel and Lavigne, 2009).

One element in semantic priming that our model did not address is post-lexical decision-making processes. Indeed, adding a decision-making module to our network may be a well-desired expansion to its core structure due to the substantial effects of decision processes in LDT (see Neely, 1991, for review). In principle, several such mechanisms could be employed (e.g., the diffusion model; Ratcliff et al., 2004). Assuming such a mechanism is operating at a late stage in processing after lexical convergence has occurred may be justified due to recent experimental evidence showing an additive effect of semantic relatedness on RT distributions (Balota et al., 2008).

In our previous studies (Lerner et al., 2012a, in press) we have shown how the dynamics in our model, beyond their contribution to sematic priming, can also form a mechanistic interpretation of previous influential models of semantic processes, such as spreading activation (Collins and Loftus, 1975) and expectancy (Becker, 1980), as well as to simulate free association norms (Nelson et al., 2004). In addition, the same model could account for the full spectrum of semantic priming findings in schizophrenic patients (Lerner et al., 2012b). Importantly, these studies have also showed how the model can be seen as advocating a new computational view regarding the distinction between automatic and controlled processes (Lerner et al., in press): whereas any processes that require transitions between representations are not automatic *per se* and can be influenced by task demands, genuinely automatic processing is based on correlations between representations. For instance, people can think of the concept *dog* while avoiding many other cognitive processes (e.g., saying “dog”; thinking of the concept *sky*; attending to the noise in the street); but they cannot think of *dog* without also partially thinking—even subconsciously—of the concept *cat*, simply due to the correlation between the representations of these concepts. Whether this definition of automatic processing can be usefully applied to other domains remains to be seen in future studies.

## Materials and methods for the neural network simulations

In this section we provide the main equations governing the network dynamics and the specific parameter values that were used in the numerical simulations of the model. Units are indicated in brackets whenever relevant. In all numeric simulations the time step represented Δ*t* = 0.66 ms.

The activity of the i-th neuron of each network at time *t*, *x*_*i*_(*t*), was a logistic function of its local input *h*_*i*_(*t*):

$$x_{i}(t)=g\left(h_{i}(t)\right),g(z)=\frac{1}{1+e^{-\frac{z}{T}}}$$

The local input obeyed the following dynamical equation:

$$\begin{array}{l} \tau_{n}h_{i}(t)=-h_{i}(t)+\sum_{j=1}^{N}J_{ij}x_{j}(t)-\lambda (\bar{x}(t)-p)-\theta \\ \;+{\left[I_{i}^{ext}(t)-\theta^{ext}\right]}_{+}+\eta_{i} \end{array}$$

Here, τ_*n*_ is the time constant of the neuron, *x*_*j*_ is the activity of the *j*-th neuron (with *x* indicating average over all neurons), *J*_*ij*_ is the connectivity weight from neuron *j* to *i*, *N* is the number of neurons, *p* is the sparseness of the representations, λ a regulation parameter which maintains stability of mean activation, and θ is a constant neuron-activation threshold, which can also be seen as global inhibition. The threshold linear function […]_+_ allows the external input to the neuron, *I*^*ext*^_*i*_(*t*), to influence the network activity only if it surpasses some constant external threshold θ^*ext*^. Finally, η_*i*_ is a noise term drawn from a Gaussian distribution with standard-deviation η_*amp*_ and temporal correlations τ_*corr*_. The temporal correlations in the noise were generated by filtering white noise using a low-pass filter, which, for two time points separated by τ ms, took the form:

$$f(\tau )=\eta_{amp}\cdot e^{-\frac{\tau }{\tau_{corr}}}$$

The synaptic depression of the connection weight between the i-th and j-th neuron obeyed (following (Tsodyks et al., 1998)):

$$J_{ij}(t)=\frac{J_{ij}^{\;\max}-J_{ij}(t)}{\tau_{r}}-Ux_{\max}x_{i}(t)J_{ij}(t)$$

with τ_*r*_ being the time constant of recovery of the synaptic efficacy, *U* the utilization of the available synaptic resources, and *x*_max_ the maximum firing rate of a neuron. *J*^max^ is the Hopfield connectivity matrix for sparse patterns (Tsodyks, 1990):

$$J_{ij}^{\max}=\sum_{\mu \;=1}^{P}\frac{\left(\xi_{i}^{\mu }-p\right)\left(\xi_{j}^{\mu }-p\right)}{Np(1-p)}$$

with *P* being the total number of memories encoded into the network, and ${\vec{\xi }}^{\mu }$ being the μ-th memory pattern. When episodic connections are added between memory ${\vec{\xi }}^{\mu }$ and ${\vec{\xi }}^{\upsilon }$, an additional term is introduced to the connectivity weights (Herrmann et al., 1993) such that: *J*^*NEW*^_*ij*_(*t*) = *J*^*OLD*^_*ij*_(*t*) + κξ^υ^_*i*_ξ^μ^_*j*_, with κ being the salience of the episodic connections compared to the semantic ones.

Reinforcement learning of the noise was implemented in the following way: at the beginning of a simulation, the noise amplitude, η_*amp*_, is initialized as a 2-dimensional vector; its first term corresponds to the noise in the semantic network at the beginning of a trial, and its second term to the noise in the semantic network after a transition has occurred (modulations after additional transitions in a trial are neglected in the current simulations; see Lerner et al., in press, for details). The actual noise amplitudes that the network “uses” during trial *n* are randomized around these base amplitude values such that each of them equals η_*amp*_(*n*) + ε(*n*), with ϵ(*n*) being an exploration parameter drawn from a Gaussian distribution with mean zero. The trial is then run using these two random noise amplitude values. At the end of each trial, each value of the noise amplitude vector is updated as follows:

$$\eta_{amp}(n+1)=\eta_{amp}(n)+\alpha \left({\bar{RT}}_{n\;-1}-RT(n)\right)\;\cdot \;\varepsilon (n)$$

Here, *RT(n)* is the Reaction Time of the lexical network to the target at the last trial, RT_*n* − 1_ the average reaction time of the previous trials (trials 1 to *n* − 1) and α the reinforcement learning rate. At the next trial, the system randomly chooses a new noise-amplitude vector centered around the two new values, and the process repeats. In order to simulate an “exploration” phase at the beginning of an experiment that decays as the experiment progresses, the variance of the exploration parameter decays with trials: ε(*n*) ~ *N*(0, *Ae*^−β*n*^).

The network parameters for the semantic and lexical networks that were used in all of the reported simulations can be found in Table 3. Parameters of the orthographic network used in Section Frequency, Degradation and Context Interactions are given here: the orthographic network contained 500 neurons with *T* = 0.01, τ_*n*_ = 7 ms, θ = 1.0. Raw connection gains (as defined in Table 3) were: Lexical-to-orthographic: 0.75; orthographic-to-lexical: 0.56/0.543 for high/low frequency. Gain of external visual input was 1.5. The Input time-delay was controlled by a time constant, τ_*ext*_, which received values of 0.66 ms/134 ms for clear/degraded targets.

**Table 3:** **The semantic and lexical network parameters**.

**Parameter**	**Semantic network**	**Lexical network**
Number of neurons, *N*	500	500
Sparseness, *p*	0.06	0.04
Correlation strength (% of overlapping active neurons out of total active neurons in a pattern)	0.1 (Strong) 0.066 (Moderate) 0.033 (Weak)	0
Neuronal gain, *T*	0.05	0.05
Neuron's time constant, τ_*n*_	7 [ms]	13 [ms]
Neuronal activation threshold, θ	0.02	0.17
Regulation parameter, λ	14.75	27.75
Maximal firing rate, *x*_*max*_	100 [spks/s]	100 [spks/s]
Utilization of synapses within each network, *U*_[within]_	0.206 [1/spks]	0 [1/spks]
Utilization of synapses between networks, *U*_[between]_	Lexical to semantic: 0.087 [1/spks]	Semantic to lexical: 0 [1/spks]
Synaptic recovery time within each network, τ_*r* [within]_	93 [ms]	–
Synaptic recovery time between networks, τ_*r* [between]_	Lexical to semantic: 1333 [ms]	Semantic to lexical: –
Input gain between networks (Raw values. Actual values were normalized by the number of pre-synaptic active neurons in a pattern)	Lexical to semantic: 2	Semantic to lexical: 0.21 (default) 0.5145 (section facilitation and inhibition in semantic priming and their relation to expectancy) 0.4977 (section frequency, degradation and context interactions)
External input gain	–	0.56
Input threshold, θ_*ext*_	1	0.25
Default noise amplitude, η_*amp*_(0) (For both initial and late noise)	Pronunciation-like: 0.05 LDT-like: 0.01	0.025
Noise temporal correlations, τ_*corr*_	17 [ms]	17 [ms]
Multiplication factor of the exploration parameter, *A*	0.06	–
Exponential coefficient of the exploration parameter, β	0.0125	–
Reinforcement-learning rate, α	0.002	–
Convergence threshold	0.95	0.95
Episodic-connections salience κ	0.0336	–

### Conflict of interest statement

The authors declare that the research was conducted in the absence of any commercial or financial relationships that could be construed as a potential conflict of interest.

## Footnotes

^1^Note, however, that this is true only on average. In fact, the exact transition probability between two concepts is a function of the whole semantic structure and is not purely determined by the degree of correlation between the pair. For a thorough discussion of this issue, see (Lerner et al., 2012a).

^2^It is important to emphasize that priming effects between associated primes and targets do not strictly depend on a transition between them; rather, they exists first and foremost because associated primes and targets often have correlated semantic representations; moreover, even when “wrong” transitions occur (that is, from the prime to a related concept other than the target) priming effects are still usually preserved because often associated primes and targets share similar related concepts. For details, please see (Lerner et al., 2012a).

^3^In fact, this is true only for facilitation. Inhibition in category-exemplar pairs increases with SOA; however, we only refer to facilitation at this point.

^4^Neely's short SOA was 250 ms, in contrast to our 150 ms. Neely's purpose in choosing this SOA was to prevent controlled processes to take part. However, 250 ms is usually considered to be on the edge of preventing such processes (and indeed, though insignificant, some inhibition is nevertheless evident in his short SOA results). In our simulations, a 250 ms SOA already permits the occasional occurrence of a transition, equivalent to the operation of expectancy. We therefore reduced this SOA to a lower value, which is more accurate giving Neely's intentions. Using an SOA of 250 ms instead of 150 ms, we found that the difference in priming between the first two SOAs is somewhat reduced, though the pattern of results remains the same as reported here.

^5^This layer should not be taken as representing real orthographic information (in which, for example, different units represent different letters). It is used simply to separate between an initial processing level where stimulus degradation makes an effect and the lexical level, which is not influenced directly by degradation. The basic idea, however, could be implemented using more realistic orthographic layers.

^6^Different targets had to be used in each priming condition because the existent association norms in Hebrew are limited in scope and, consequently, it was impossible to find all three types of primes for each target

^7^Some small differences from the text may be evident, due to rounding

^8^Only subjects-analysis is reported. As noted by Raaijmakers et al. (1999) and McNamara (2005), and in contrast to a common practice in the field, jointly reporting subjects- and items-analysis is wrong, and, moreover, when a counterbalanced design is used—such as in our experiments—subjects-analysis is the only required statistics.

^9^In the simulation, however, we used SOAs of 100 and 200 ms instead of the 50 and 150 ms SOAs of the human experiment, to ensure correct convergence of the network to the prime, as such convergence is not always achieved for 50 ms. Nevertheless, the principle mechanism that we demonstrate in this simulation—namely, the lack of semantic transitions at very short SOAs—is not affected by this small modification.
